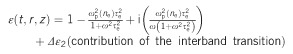# Electrons dynamics control by shaping femtosecond laser pulses in micro/nanofabrication: modeling, method, measurement and application

**DOI:** 10.1038/lsa.2017.134

**Published:** 2018-02-09

**Authors:** Lan Jiang, An-Dong Wang, Bo Li, Tian-Hong Cui, Yong-Feng Lu

**Affiliations:** 1 Laser Micro/Nano-Fabrication Laboratory, School of Mechanical Engineering, Beijing Institute of Technology, Beijing 100081, China; 2 Department of Mechanical Engineering, University of Minnesota, Minneapolis, MN 55455, USA; 3 Department of Electrical Engineering, University of Nebraska-Lincoln, Lincoln, NE 68588-0511, USA

**Keywords:** electrons dynamics control, femtosecond laser, micro/nano fabrication, pulse shaping

## Abstract

During femtosecond laser fabrication, photons are mainly absorbed by electrons, and the subsequent energy transfer from electrons to ions is of picosecond order. Hence, lattice motion is negligible within the femtosecond pulse duration, whereas femtosecond photon-electron interactions dominate the entire fabrication process. Therefore, femtosecond laser fabrication must be improved by controlling localized transient electron dynamics, which poses a challenge for measuring and controlling at the electron level during fabrication processes. Pump-probe spectroscopy presents a viable solution, which can be used to observe electron dynamics during a chemical reaction. In fact, femtosecond pulse durations are shorter than many physical/chemical characteristic times, which permits manipulating, adjusting, or interfering with electron dynamics. Hence, we proposed to control localized transient electron dynamics by temporally or spatially shaping femtosecond pulses, and further to modify localized transient materials properties, and then to adjust material phase change, and eventually to implement a novel fabrication method. This review covers our progresses over the past decade regarding electrons dynamics control (EDC) by shaping femtosecond laser pulses in micro/nanomanufacturing: (1) Theoretical models were developed to prove EDC feasibility and reveal its mechanisms; (2) on the basis of the theoretical predictions, many experiments are conducted to validate our EDC-based femtosecond laser fabrication method. Seven examples are reported, which proves that the proposed method can significantly improve fabrication precision, quality, throughput and repeatability and effectively control micro/nanoscale structures; (3) a multiscale measurement system was proposed and developed to study the fundamentals of EDC from the femtosecond scale to the nanosecond scale and to the millisecond scale; and (4) As an example of practical applications, our method was employed to fabricate some key structures in one of the 16 Chinese National S&T Major Projects, for which electron dynamics were measured using our multiscale measurement system.

## Introduction

Because of their ultrashort irradiation periods and ultrahigh intensities, femtosecond laser pulses in some aspects fundamentally change the laser-material interactions mechanisms compared with long laser pulses, which has created wide-range and exciting new possibilities in micro/nanoscale fabrication^1, 2, 3^.

The ultrahigh intensity makes femtosecond laser-material interactions a strongly nonlinear process^4, 5^. Linear ionization generally dominates in free electron generations for continuous or long-pulse laser processing of wide bandgap materials^6^. The intensities of femtosecond lasers can easily exceed 10^12^ W cm^−2^, thus nonlinear ionization mechanism such as avalanche ionization (~10^12^ W cm^−2^), multiphoton ionization (~10^13^–10^14^ W cm^−2^), and tunnel ionization (>10^15^ W cm^−2^; Ref. 3), can occur in the femtosecond laser fabrication processes. The nonlinear ionizations are almost independent on the initial defects of the target materials. Hence, femtosecond laser ablation is deterministic and reproducible^7^, and almost any material can be machined using a femtosecond laser, including metals^8, 9, 10^, semiconductors^11, 12^, dielectrics^13, 14^, polymers^15^, two-dimensional materials^16, 17, 18, 19, 20^, ultrahard materials^21^ and biological tissues^22, 23^. In addition, ionization mechanism can be adjusted by changing femtosecond laser energy and its temporal/spatial distribution to control laser-material interactions^24^.

The ultrashort irradiation period of a femtosecond laser also makes femtosecond laser-material interactions a strongly nonequilibrium process^3, 25^. The duration of a femtosecond laser pulse is much shorter than the electron-lattice energy relaxation time (10^−10^−10^−12^ s). Therefore, laser energy absorption is completed before the lattice changes, resulting in a significantly nonequilibrium state between electrons and lattices. Hydrodynamic motion and heat conduction through lattices are negligible during the femtosecond pulse duration. Thus, recast, thermal damage (microcracks) and heat-affected-zone are greatly reduced^2^. Because of the nonequilibrium laser-material interactions, including phase change and material removal, are essentially determined by laser-electron interactions^26, 27^. Hence, femtosecond laser fabrication must be improved by controlling electron dynamics during the fabrication process, which poses a challenge for measuring and controlling at the electron level during fabrication processes.

Pump-probe spectroscopy, which has been widely used in fields including physics^28, 29, 30, 31, 32, 33^, chemistry^34, 35, 36, 37^ and materials science^38, 39, 40^, presents a viable solution for detecting electron dynamics. The basic mechanism involves splitting one femtosecond pulse into two sub-pulses. One sub-pulse is the pump pulse and the other is the probe pulse, and the time delay between them can be precisely controlled by adjusting the optical path difference. On the basis of this principle, Zewail^41^ and other pioneers developed the discipline of femtochemistry, which elucidates ultrafast chemical reaction processes at the electron level^42, 43, 44^. Their research demonstrates how chemical bonds change and electrons transfer during chemical reactions^45, 46^. Recently, attosecond laser pulses have been used to probe electron dynamics in greater detail^47, 48, 49^, and even the electronic charge distribution within molecules has been detected^34, 50, 51^. In addition, electron dynamics during laser-material interactions have been captured through the pump-probe technique. For instance, researchers have imaged electron excitation, electron spatiotemporal distribution and electron decay on the femtosecond scale^52, 53, 54^. Furthermore, the electron collision time and laser-induced plasma lifetime can be determined using pump-probe measurement^55, 56, 57^. The information on electrons measured from experiments may contribute to theoretical comprehension and prediction.

Many innovations in measurement technologies could also be converted to new methods in fabrication technologies. In the pump-probe experiments, the observation of electron dynamics is achieved by analyzing the probe pulse disturbed by electrons. By increasing the energy of the probe laser, the disturbing process can be transformed into certain control if it is purposely designed. Much more complex pulses other than merely two pulses can be designed to control the electron dynamics. Recent developments of optical devices substantially enhanced the capability to shape laser pulses. The amplitude^58, 59, 60, 61, 62, 63^/phase^64, 65^/polarization^66, 67^/frequency^68, 69^ can be easily manipulated in both temporal and spatial domains. By temporally/spatially shaping the femtosecond laser, the local transient electron dynamics can be precisely controlled. Many studies have demonstrated that properties of atoms and molecules can be controlled by temporal/spatially shaped ultrafast laser pulses^70, 71, 72^. For example, using a shaped ultrafast laser pulse, atoms can be selectively ionized^73, 74, 75^; spin states can be dynamically controlled^76, 77, 78, 79, 80^; molecular ground state rotational dynamics and vibrational modes can be manipulated^81, 82^; chemical reactions can be controlled^83, 84^ and X-ray line emission from plasma under the femtosecond pulse can be significantly enhanced^85, 86, 87^. In the field of ultrafast laser fabrication, Herman and Ilday *et al*^5, 88, 89, 90^ demonstrated the heat accumulation effect can be controlled by harnessing high-repetition-rate burst trains of ultrafast laser pulses, achieving significant benefits compared with unshaped pulses. Xu *et al*^24, 91, 92^ revealed the conceptual significance of controlling coherent phonon dynamics for controlling phase change by specifically designed ultrafast pulse trains with different pulse delays. Gamaly^3^ reported controlling over the ablation rate and phase state of laser-produced plume by using temporal/sptial pulse shaping. Sheppard and Wilson^93^ produced the Bessel beam with an annular lens, and Marcinkevičius *et al*^94^ and Courvoisier *et al*^95, 96, 97^ obtained some micro/nanostructures by spatially shaping ultrafast laser pulses.

Although various results have been obtained to control the laser-material interactions, electron dynamics have not been extensively studied or deliberately controlled, which is vital for laser micro/nano fabrication. In femtosecond laser micro/nano fabrication process, photons are mainly absorbed by electrons, and the subsequent energy transfer from electrons to ions is of picosecond order (10^−12^−10^−10^ s). Hence, lattice motion is negligible within the femtosecond pulse duration, and femtosecond photon−electron interactions are the only factor to be considered, which dominates the subsequent fabrication processes (10^−10^–10^−3^ s). Therefore, femtosecond laser fabrication must be improved by controlling localized transient electron dynamics. The key challenge is to measure and control at the electron level during fabrication processes. Pump-probe spectroscopy presents a viable solution, which can be used to observe electron dynamics during a chemical reaction. In the pump-probe experiments, the observation of electron dynamics is achieved by analyzing the probe pulse disturbed by electrons. By increasing the energy of the probe laser, the disturbing process can be transformed into certain control if it is purposely designed. Therefore, we propose the core idea of electrons dynamics control (EDC; Supplementary Movie 1, Supplementary Information): By shaping the amplitude, phase and polarization of femtosecond pulses in temporal and spatial domains, we are able to control photon–electron interactions, and then to control the localized transient electron dynamics (including electron density, temperature and excited-state distribution), and further to modify localized transient materials properties, and then to adjust material phase change, and finally to implement the novel fabrication method. We have devoted our efforts on using EDC to improve femtosecond micro/nano fabrications for more than a decade. This review summarizes our recent progresses based on EDC by shaping femtosecond pulses in micro/nanofabrication:

1. Theoretical modeling for EDC feasibility: four models were developed, which consists of the *ab initio* calculations for electron dynamics^98, 99, 100, 101, 102, 103, 104, 105, 106, 107^, a revised molecular dynamics simulation for phase change^108, 109^, a plasma model for ionization processes^110, 111, 112, 113, 114, 115^ and an improved two-temperature model for energy transport^116, 117, 118^.
2. Experiments for EDC validation: By shaping a femtosecond pulse in temporal or spatial domains, the photon-electron interactions can be controlled to adjust the localized transient electron dynamics to implement the novel fabrication methods. Various experiments were conducted to validate the effectiveness of EDC. Based on EDC, we proposed to:
  - control the localized transient electron density to induce resonance absorption, by which microchannel processing efficiency was increased by 56 times and the maximum aspect ratio was extended by three times^119, 120^;
  - modify free electron density and the corresponding photon absorption efficiency, then modulate material properties, by which laser-assisted chemical etching rate was enhanced by 37 times^121^;
  - adjust electron generation on fabricated material surfaces, through which the periods, orientations and structures of the surface ripples can be effectively adjusted^122, 123^;
  - control electron density and its distribution, through which we obtained controllable micro/nano hierarchical structures on material surfaces and enhancement factors of surface-enhanced Raman scattering (SERS) up to 1.1 × 10^9^ (Refs. 124, 125, 126);
  - control electron density distribution using temporally shaped femtosecond laser pulse to modify chemical and physical properties of materials, by which polymorphous Au-MoS_2_ hybrids were prepared^127^;
  - adjust the phase change mechanism by changing photon-electron interactions, which reduced the recast layer thickness by 60% (Ref. 128);
  - manipulate electron density distribution using spatially modulated femtosecond laser pulse, by which deep subwavelength (∼1/14 of the laser wavelength) and high conductivity (∼1/4 of the bulk gold) nanowires were fabricated in the open air^129^.
3. Measurements for EDC fundamentals: A multiscale measurement system (from femtosecond scale to millisecond scale) was proposed and developed for understanding the electron dynamics during femtosecond laser ablation. It comprises a pump-probe shadowgraph imaging technique^130, 131^, time-resolved plasma photography^132, 133^, laser-induced breakdown spectroscopy (LIBS)^134^ and commercialized fast imaging device (CCD). We reveal the multiple time scale fundamentals during femtosecond laser-material interactions, including the femtosecond-scale propagation of a laser pulse, picosecond-scale generation/evolution of laser-induced plasma, nanosecond-scale plasma ejection/expansion, and millisecond-scale hole formation.
4. Applications for the EDC method: Our proposed method was used to fabricate some key structures. Using spatially-shaped femtosecond pulses, we optimized electron density distribution in plasma at the focus point, and then manipulated plasma expansion and phase change, which was used to drill microholes with a diameter of 1.6 μm and an aspect ratio of 330:1 (Refs. 132, 135). Furthermore, we revealed the forming mechanisms of the high-quality and high aspect-ratio microholes using the multiscale time-resolved measurement system^131^.

## Modeling for EDC feasibility

### Modeling method

Femtosecond photon–electron interactions dominate the entire nonequilibrium and nonlinear laser fabrication processes, including the absorption of laser energy by electrons, energy transfer from electrons to lattices, plasma generation, phase change^136, 137, 138^ and material modification. These interactions range from nanometers to millimeters spatially and from femtoseconds to microseconds temporally. Although a growing body of experimental observations exists, a comprehensive model remains unavailable. Such a model is essential for revealing the fundamental science underlying ultrafast laser-material interactions. According to the applicable time and space scales of each involved process, four fundamental models were used:

1. The *ab initio* model based on the time-dependent density functional theory (TDDFT) was employed to understand initial nonlinear laser radiation absorption through photon-electron interactions^98, 99^.

In TDDFT, the fundamental variable is not the many-body wave function in quantum mechanics, but the electronic density. This time-dependent electron density is determined by solving an auxiliary set of noninteracting Kohn-Sham equations, where the laser fields are treated as time-dependent spatial uniform external vector potentials. Describing the motion of the electrons in the system, the time-dependent Kohn-Sham equation for single particle orbitals is


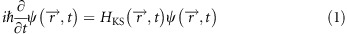



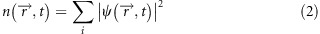


where 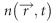 is the electron density and 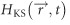 is the Kohn-Sham Hamiltonian given by


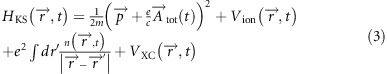


where 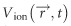 is the electron-ion potential and 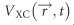 is the exchange-correlation (XC) potential.The time evolution of the wave function over a short period, Δ*t*, can be approximately calculated as follows:


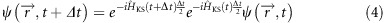


2. The molecular dynamics model was employed to reveal the phase change resulting from electron-ion interactions^109^. In the molecular dynamics model, Newtonian equations of motion of a set of N particles are solved to describe the phase change process.


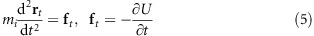


where **r**_*i*_ is the coordinate of the *i*th atom, and **f**_*i*_ is the force acting on the *i*th atom that is usually derived from potential energy *U*(*r*) (Ref. 139):


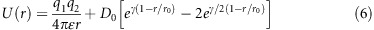


3. We proposed the plasma model with quantum treatment to investigate plasma generation and changes (ionization and recombination)^110, 113, 114, 115^.In the plasma model^114, 115^, the free electron density distribution in dielectrics under a femtosecond laser pulse is obtained by solving the Fokker−Planck Equation:


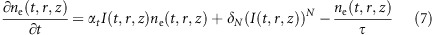


where *t* is time, *r* is the distance to the Gaussian beam axis, *z* is the depth from the surface of the bulk material, *n*_e_*(t,r,z)* is the free electron density, *τ* is the free electron recombination time, *α*_*i*_ is the impact ionization constant, and *δ*_N_ is the cross-section of *N*-photon absorption.The optical properties of ionized dielectrics are calculated using the Drude model. The spatial and temporal dependence of the complex dielectric function for the plasma is expressed as:


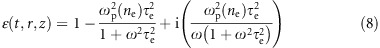


where 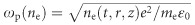 is the plasma frequency and *τ*_e_ is the free electron relaxation time, calculated as, follows by applying formulas derived from the Boltzmann transport Equation:


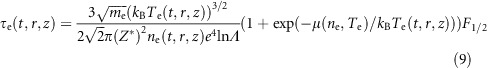


where Z* is the ionization state, *m*_e_ is the electron mass, *k*_B_ is the Boltzmann constant, *F*_1/2_ denotes the Fermi-Dirac integrals, *μ* is the chemical potential and *ln Λ* is the Coulomb logarithm.In addition, the wave-particle duality of photon is also considered to predict the formation mechanism of laser-induced periodic surface structures^110^.

4. We proposed the improved two-temperature model with full-run quantum treatment to predict the electron and lattice temperature distributions^116, 117^. In the improved two-temperature model, electron and lattice temperatures are given by


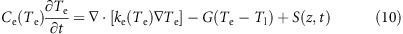



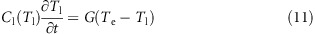


where *T*_e_ is the electron temperature, *T*_l_ is the lattice temperature, *C*_e_ is the electron heat capacity, *C*_l_ is the lattice heat capacity, *k*_e_ is the electron heat conductivity, *G* is the electron-lattice coupling factor, and *S* is the laser source term.

**Figure 1:**
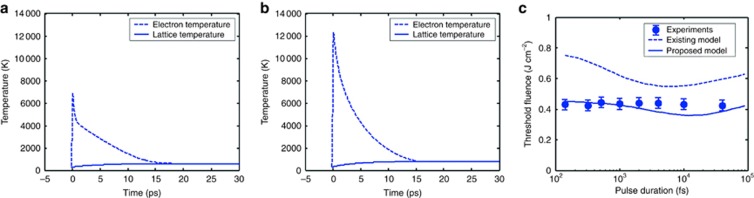
The calculated electron and phonon temperatures of 200 nm gold film irradiated by a 140 fs, 1053 nm pulse at 0.2 J cm^−2^ by (**a**) the classical model and (**b**) the improved model. (**c**) The predicted damage threshold fluences of 200 nm gold film processed by a 1053 nm laser at different pulse. Reproduced from Ref. 117 (with the permission of SPIE).

The two-temperature model was improved by us as follows^116^: (1) using the Fermi distribution, the heat capacity of the free electrons was calculated; (2) free-electron relaxation time and electron conductivity were determined using a quantum model derived from the Boltzmann transport equation for dense plasmas; and (3) the free-electron heating and interband transition were taken into account using a modified Drude model as Equations (8) and (9) to calculate the reflectivity and absorption coefficient. Table 1 compares the estimates of optical and thermal properties between classical and improved two-temperature models. Figure 1 demonstrates the differences in (a, b) electron and phonon temperatures between the classical approach and the improved model. The improved model significantly increases the prediction precisions of the damage thresholds compared with the classical model, as shown in Figure 1c^117^.

The four theoretical models are suitable for systems with different temporal and spatial scales and must be employed in combination to compensate for each other’s limitations. In our improved two-temperature model, the electron density is assumed to be constant. Consequently, the model is not suitable when the bound electrons in non-metals are substantially ionized by a femtosecond laser pulse. The plasma model is proposed to consider the ionization process in nonmetals, but material ablation is assumed to commence when the free electron density reaches the critical density without the consideration of phase changes, for example, melting, vaporization, Coulomb explosion and electrostatic repulsion. Moreover, the molecular dynamics can be used to identify the relative roles of different phase change mechanisms. However, the interatomic potential remains constant in the molecular dynamics model, even though it in fact changes dramatically during strong ionizations. The *ab initio* method is therefore used to describe a system with no parameterization, which facilitates the investigation of processes whose mechanisms are not fully understood. In a typical *ab initio* method, the potential energy for a particular electronic state is defined by the electronic Schrödinger equation. However, the computational demand of *ab initio* calculation is substantial, and impact ionization is not considered because the theory is based on orbitals that interact only through the mean field. The exact time-dependent xc potential and the functional for physical observables are still being studied.

On the basis of the aforementioned factors, during femtosecond laser-material interactions, the target material can be conceptually divided into three systems: the electron system, atom or molecule system and plasma system outside the bulk material. The *ab initio* model can be applied to sub-nanometers sampling areas to determine the ionization mechanisms^102^ and multiphoton absorption cross section^104^. The revised molecular dynamics can be employed to determine the phase change mechanisms in areas of tens to hundreds of nanometers within an electric field formed by the plasma system outside the bulk material, which is calculated using our plasma model^108^. The plasma model can be used to describe the electron system for photon absorption and plasma generation (ionization) and recombination before the lattice actually changes^110, 114^. Our improved two-temperature model can be employed to calculate energy transport through electron-phonon interactions^116, 117^. Using the models, we validated the concept of EDC and predicted its potentials in ultrafast laser micro/nanofabrication^88, 89, 90, 91, 92, 93, 94, 95, 96, 97, 98, 99, 100, 101, 102, 103, 104, 105, 106, 107^.

### Simulation results

**Figure 2:**
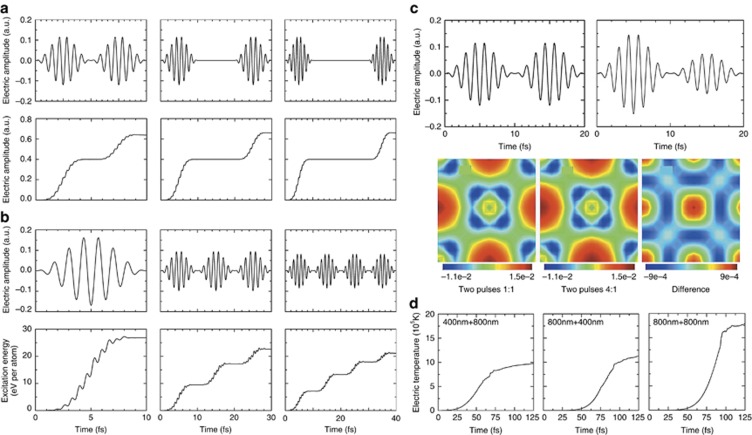
Schemes of electrons dynamics adjusted by shaped femtosecond laser pulses. Electric fields of the applied laser pulse and time-dependent excited electrons of diamond with different (**a**) pulse delays^99^ and (**b**) sub-pulse numbers^99^. (**c**) Electric fields of the applied laser pulse (top panel) and the electron density change of diamond from that in the ground state after laser termination with different pulse energy ratios^99^. (**d**) Time-dependent electron temperature of fused silica with different pulse dual-wavelengths^111^. Reproduced from Ref. 99 (with the permission of IOP) and Ref. 111 (with the permission of AIP publishing).

In this section, we report several representative simulation results. As shown in Figure 2, the time-dependent electrons dynamics in materials irradiated by temporally shaped femtosecond laser pulse train have been calculated based on TDDFT^99^ and the improved two-temperature model^111^. The simulation results indicated that increasing pulse delay from 10 to 30 fs markedly increased the number of excited electrons and absorbed energy (Figure 2a). However, the excitation energy decreased as the sub-pulse number per train increased from 1 to 4 (Figure 2b). Furthermore, the shaped pulse energy and wavelength distribution were also determined to be key parameters for changing the electron density and temperature (Figure 2c and 2d). The results indicated that localized transient electrons dynamics, including the number of excited electrons, excitation energy, electron density distribution, and electron temperature, can be controlled by shaping femtosecond pulses (pulse delay, sub-pulse number, pulse energy ratio and dual-wavelength distribution).

**Figure 3:**
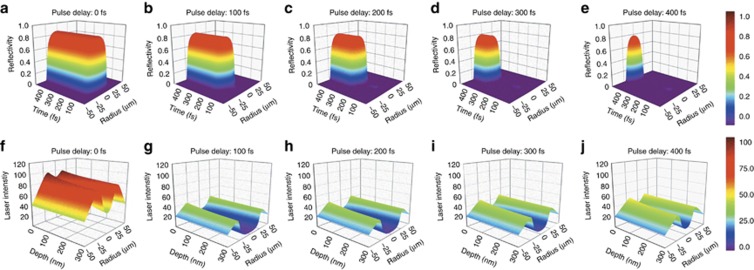
Simulation of materials properties adjusted by varying pulse delay within a femtosecond laser pulse train. (**a**–**e**) Reflectivity of the material surface and (**f**–**j**) the corresponding peak laser intensity distribution at different pulse delays.

We theoretically demonstrated that localized transient materials properties can be effectively modulated through localized transient EDC by temporally shaping femtosecond laser pulses (Figure 3). The reflectivity and corresponding peak laser intensity distribution were completely changed by varying the pulse delays. Moreover, the electron density was adjusted by varying the pulse delay, and reflectivity decreased as the pulse delay increased because of the free electron density was lower. Thus, the original laser intensity distribution was considerably reshaped, providing substantial control over the energy absorption process. The results demonstrated that by controlling localized transient electron dynamics through shaping femtosecond pulses, the localized transient material properties, such as the reflectivity and absorption coefficient, can be modified.

**Figure 4:**
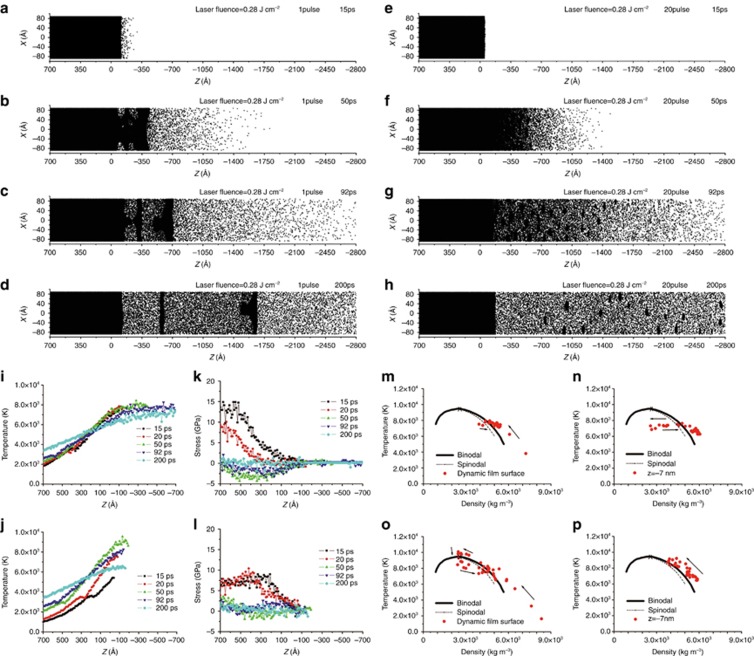
Schemes of phase change controlled by varying the pulse delay in a fs pulse train^108^. Snapshots of nickel thin films irradiated by femtosecond laser (**a**–**d**) single pulse and (**e**–**h**) 20 pulse trains with the total fluence of 0.28 J cm^−2^, where *X* is in the direction of Ni (100) surface and *Z* is in the direction of laser irradiance. Lattice temperature and stress distributions at different times for (**i** and **k**) the single pulse and (**j** and **l**) the 20 pulse trains. Time evolution of the system in the *ρ*-*T* plane for different regions for (**m** and **n**) the single pulse and (**o** and **p**) the 20 pulse trains. Arrows indicate the time evolution. Reproduced from Ref. 108 (with the permission of AIP publishing).

Meanwhile, we studied the phase change mechanisms in materials such as nickel thin films under irradiation by femtosecond laser pulse trains by using molecular dynamics simulations and the two-temperature model^108^. The theoretical simulation revealed that more and smaller high-quality uniform nanoparticles can be obtained on the nickel thin film by femtosecond laser pulse trains compared with conventional pulses (Figure 4a–4h). The use of pulse train reduced the electron temperature and electron thermal conductivity dramatically because of the lower intensity of the sub-pulses or higher transient surface temperatures, which left the absorbed energies deposited mainly within the nanoscale layers of the dynamic film surface (Figure 4i and 4j). By designing the pulse train, smaller film compressive stresses and tensile stresses can be obtained, which reduced microcracks (Figure 4k and 4l). Furthermore, a transition from phase explosion to critical point phase separation (Figure 4m–4p) enabled small uniform nanoparticle generation. The modulated phase change mechanism was considered the main factor for the morphology control. Both the compressive and tensile stresses can be reduced by the pulse trains, leading to the critical point phase separation within the uppermost films and no liquid-vapor phase separation within the subsurface films when total laser fluence was slightly above the volumetric phase change threshold fluence of single pulse ablation. Therefore, the results demonstrated that the phase change mechanism can be controlled by carefully designing the pulse train parameters.

**Figure 5:**
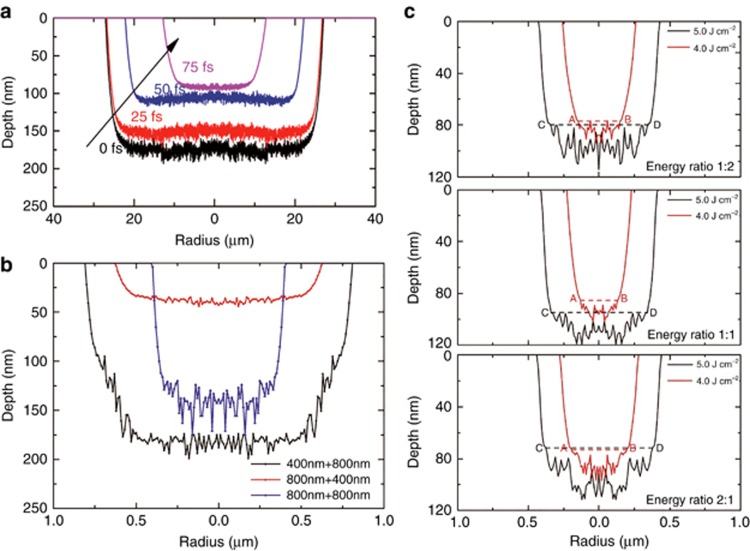
Schemes of ablation crater shape controlled by shaped femtosecond laser pulse^110, 111, 112^. (**a**) Ablation crater shapes created by femtosecond pulse trains consisting of double pulses with different pulse delays at a total fluence of 5 J cm^−2^ and central wavelength of 780 nm. (**b**) Ablation crater shapes created by femtosecond laser pulse trains with different wavelength composition at the total fluence of 5.0 J cm^−2^ and the pulse delay of 50 fs. (**c**) Ablation crater shapes created by 800 nm femtosecond pulse trains consisting of double pulses with three different energy ratios at the pulse delay of 50 fs. Reproduced from Ref. 110 (with the permission of IOP), 111 (with the permission of AIP publishing) and 112 (with the permission of Springer).

The ablation shape modulated by designed femtosecond pulse trains on fused silica was validated using our plasma model^110, 111, 112^. The crater shapes of the fused silica and the spacing of the subwavelength ripples differed greatly among sub-pulses with different delay times, wavelengths and energy distributions. The ablation depths were approximately 170, 145, 102 and 91 nm for pulse delays of 0, 25, 50 and 75 fs, respectively (Figure 5a). The ablation depths were approximately 186, 43 and 139 nm, and the ablation radii were approximately 860, 630 and 410 nm for pulse trains of 400+800, 800+400 and 800+800 nm, respectively (Figure 5b). For 4.0 J cm^−2^, the depths of the microholes were 77, 84 and 71 nm (depth from the surface to the position of line AB), and the corresponding radii of the ablation craters were 250, 160 and 270 nm for the energy ratios of 1:2, 1:1 and 2:1, respectively; For 5.0 J cm^−2^, the depths of the microholes were 80, 95 and 70 nm (depth from the surface to the position of line CD), and the corresponding radii of the ablation craters were 430, 410 and 440 nm for the energy ratios of 1:2, 1:1 and 2:1, respectively (Figure 5c). Calculations based on the plasma model revealed that the time-dependent free electron density differed substantially among laser pulses with different parameters. Changing the ionized electron density distributions significantly modified the optical and thermal properties of the material. This interaction process greatly altered ablation shape and subwavelength ripple. The results indicated that by controlling femtosecond pulses trains, the final material modification can be improved.

The model simulations validate theoretical feasibility of EDC, providing a theoretical prediction and guide for practical experiments and applications. Furthermore, our models and subsequent multiscale measurements can form a mutually-supporting system. According to the theoretical prediction, a novel fabrication method based on temporally and spatially shaped pulse trains was proposed. Localized transient electron dynamics and the corresponding material properties can be actively controlled based on the proposed method. We discuss several pieces of experimental evidences in detail in the following sections.

## Experiments: EDC-based novel fabrication method

To validate the proposed method of EDC-based fabrication method through temporal and spatial shaping of femtosecond laser pulses, many experiments have been conducted^119, 120, 121, 122, 123, 124, 125, 126, 127, 128^. By designing temporally shaped pulse trains, we can control the localized transient electron density to induce resonance absorption; thus, high efficiency fabrication can be achieved^119, 120^. We can adjust the phase change mechanism from thermal phase change to nonthermal phase change; subsequently, high-quality fabrication can be performed^128^. In addition, we can modify the free electron density, the corresponding photon-absorption efficiency and the material properties, enabling us to further control the chemical reaction^121^. We can adjust the electron density and its distribution so that the periods, orientations and structures of the surface ripples can be effectively modulated^122, 123, 140^ and a high sensitivity of SERS substrate (controllable micro/nano hierarchical structures on materials’ surfaces) can be achieved^124, 125^. Furthermore, we can control the electron density distribution to induce surface chemical reduction activity in materials, enabling us to further modify the chemical and physical properties of the materials^127^. By spatially shaping a femtosecond laser, a metal nanowire with a super sub-diffraction-limit precision (1/14th of the wavelength) can be achieved^129^.

### Femtosecond laser temporal pulse shaping

**Figure 6:**
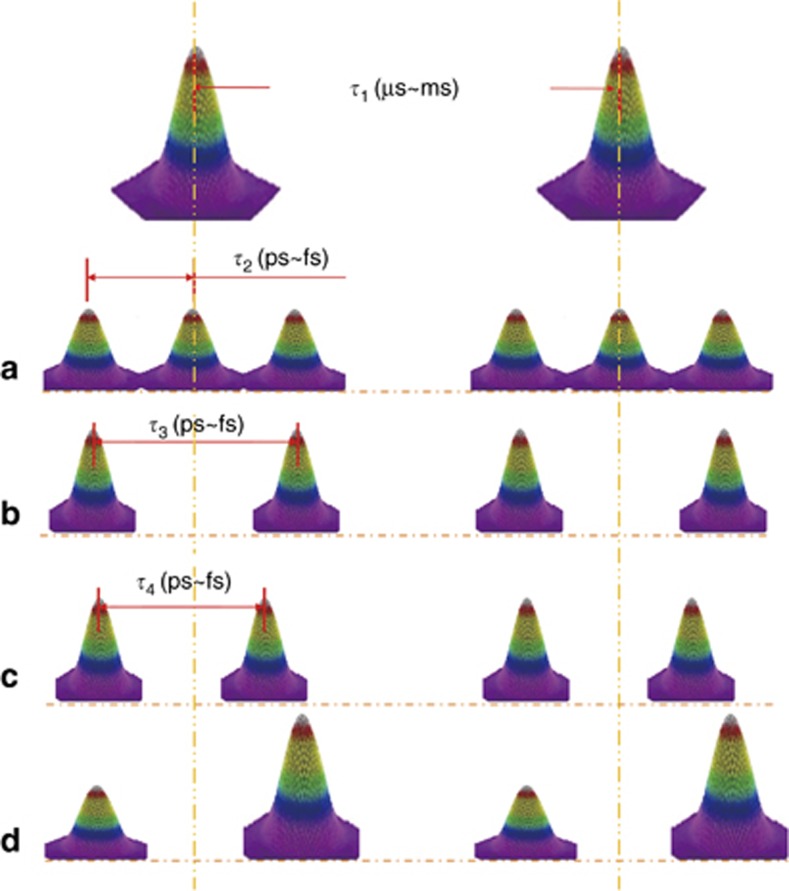
Temporally shaping femtosecond pulses: (**a**) a conventional femtosecond pulse is temporally shaped into a pulse train; (**b**) the number of sub-pulses within a train can be controlled; (**c**) the delay between sub-pulses can be controlled; (**d**) the energy ratio of sub-pulses can be controlled.

During femtosecond laser fabrication, most of the photon energy is initially absorbed by electrons. However, conventional femtosecond laser pulses are separated by a time scale that ranges from microseconds to milliseconds (Figure 6), which is much longer than the time scale of electron-lattice coupling (typically a few picoseconds to tens of picoseconds). Temporal pulse shaping enables sub-pulse generation with a pulse delay shorter than the characteristic time scale of electron-lattice coupling so that we can control femtosecond laser photon–electron–phonon interactions. As Figure 6a shows, a conventional femtosecond laser pulse can be split into several sub-pulses with a delay, which is called the pulse train. The separation between the sub-pulses occurs in the time scale—tens of femtoseconds to several picoseconds—which is generally similar to the characteristic time scale of electron-lattice coupling. Before the photon energy that is absorbed by the electrons transfers to lattice, the subsequent sub-pulses continuously interact with the materials so that controlling ultrafast photon-electron-phonon interactions is possible. Moreover, a conventional femtosecond laser single pulse can be shaped into almost any arbitrary pulse shapes; for example, (1) a pulse can be split into a pulse train with different numbers of sub-pulses (Figure 6b); (2) the delay between the sub-pulses can be controlled (Figure 6c); and (3) the energy ratio of the sub-pulses can be controlled (Figure 6d). By shaping a femtosecond laser pulse in temporal domains to obtain a specific pulse shape, we can control the photon-electron interactions, which allow us to control the localized transient electron dynamics, including electron density, temperature, excited state distribution and further modify localized transient material properties, adjust material phase changes and ultimately implement the novel fabrication methods.

#### *Experimental setup for temporal pulse shaping*

**Figure 7:**
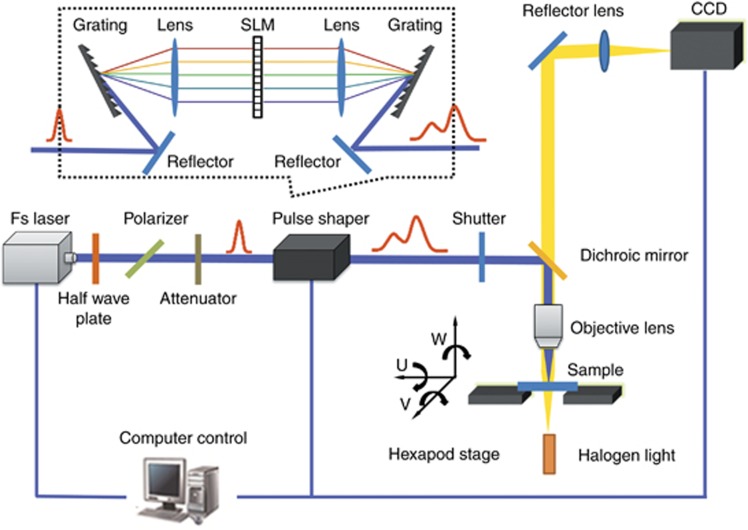
Schematic of the experimental setup for temporal shaping of femtosecond pulses. Reproduced from Ref. 141(with the permission of Springer).

The schematic of the experimental setup for temporal shaping of femtosecond laser pulses is shown in Figure 7 (Ref. 141). An amplified Ti: sapphire laser system (Spectra Physics Inc., Santa Clara, CA, USA) is used to generate 35 fs (full width at half maximum) linearly polarized laser pulses on a central wavelength of 800 nm with a repetition rate of 1 kHz. In addition, the pulse energy can be up to 3.5 W and can be continuously adjusted by combining a half-wave plate with a polarizer. Pulse energy can also be reduced to the desired values according to specific experimental conditions by using a neutral density (ND) filters. Temporal shaping of the femtosecond laser pulses can be achieved by using a commercial 4f-configuration-based pulse shaper (BSI MIIPS BOX 640, Biophotonic Solutions, Inc., East Lansing, MI, USA), which allows us to split each conventional single pulse into a pulse train and control the number of sub-pulses, delay between sub-pulses and energy ratio of the sub-pulses within a pulse train. The irradiation time (that is, number of pulse bursts) is precisely controlled by using an electromechanical shutter. The sample is mounted on a computer-controlled, six-axis translation stage (M-840.5DG, PI, Inc., Karlsruhe, Germany) with a positioning resolution of 1 μm. The entire fabrication process can be observed by using a charge-coupled device (CCD) camera along with a white-light source irradiate on the sample surface.

#### *High-efficiency fabrication by temporal pulse shaping based on EDC*

**Figure 8:**
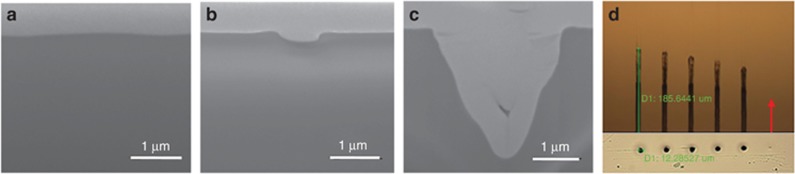
Holes machined using (**a**) five 355-nm nanosecond pulses (duration of 30 ns, energy of 0.16 μJ)^119^, (**b**) five 800-nm femtosecond pulses (duration of 120 fs, energy of 0.12 μJ)^119^, (**c**) five femtosecond-nanosecond pulse pairs^119^, (**d**) an 800 nm femtosecond laser double-pulse train (duration of 50 fs, energy of 20 μJ) ^120^. Reproduced from Ref. 119 (with the permission of OSA) and Ref. 120 (with the permission of OSA).

By designing femtosecond laser pulse trains on the basis of EDC, we can control the localized transient electron density to induce resonance absorption between laser and its generated plasma; thus, high efficiency fabrication can be achieved^119, 120^. As illustrated in Figure 8a, when five 355 nm nanosecond laser pulses irradiated on fused silica, no apparent damage was observed because the photon energy (∼3.5 eV) of the 355 nm wavelength is lower than the bandgap (∼8.9–9.3 eV) of fused silica, resulting in little absorption of laser energy^120^. For five femtosecond laser pulses, a shallow hole was machined (Figure 8b), because of the low-efficient 800 nm (∼1.55 eV) photon absorption through multiphoton ionization in wide bandgap dielectrics. In Figure 8c, femtosecond-nanosecond dual-beam laser manufacturing revealed that a much higher fabrication efficiency (that is, a 50.7-fold enhancement in material removal volume) was obtained. This enhancement was attributed to the high free electron density generated by the femtosecond laser pulses, which leading to the significantly-increased absorption of the nanosecond laser pulses energy. However, the femtosecond-nanosecond dual-beam system was complex, and the quality of the as-fabricated holes could not be guaranteed. To date, temporally shaped femtosecond laser double-pulse train was used to manufacture high-quality microholes with high-efficiency^119^. Through temporal pulse shaping, free electron density can be adjusted to be around the critical point, at which the laser frequency equal to the plasma frequency, nearly optimizing to the resonance absorption so that the fabrication efficiency is enhanced 56 folds and the aspect-ratio is enhanced 3 fold (Figure 8d).

#### *Chemical etching controlled by temporal pulse shaping based on EDC*

**Figure 9:**
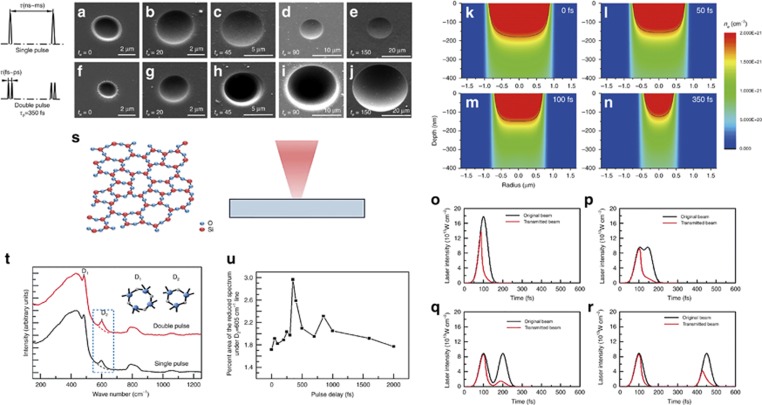
Morphology evolution of the sample surface exposed by (**a**–**e**) a femtosecond laser single pulse; (**f**–**j**) femtosecond laser double pulses at different stages of the etching process, the pulse delay is 350 fs. The SEM images have varying scale bars. (**k**–**n**) Simulation of the free electron density distributions and (**o**–**r**) center laser intensity distributions in fused silica irradiated by femtosecond double pulses at different pulse delays. (**s**) Schematic diagram of the manufacturing and Si-O bond structure. (**t**) Normalized Raman spectra of modified regions irradiated using femtosecond laser single and double pulses (the pulse delay is 350 fs) in fused silica. Dashed lines below the D2 peaks are baselines used in the peak area measurement in **u**. Inset is the schematic diagram of 4- and 3-membered ring structures. (**u**) Percent area of the total reduced Raman spectrum under the D2 line versus different pulse delays. The femtosecond laser with wavelength of 800 nm, duaration of 50 fs and repetition rate up to 1 KHz. The laser fluence is fixed at 9.46 J cm^−2^ in all experiments and the energy distribution ratio is 1:1. Reproduced from Ref. 121 (with the permission of NPG).

By designing femtosecond pulse trains on the basis of EDC, the free electron density and corresponding photon-absorption efficiency can be modified. Thus, the material properties can be modified to improve the etching rate of fused silica^121^ (Figure 9). Compared with conventional femtosecond laser pulses, femtosecond laser double-pulse trains achieved a 37-fold enhancement in the laser-assisted chemical etching rate. Simulations indicated that by optimizing the pulse delay between the two sub-pulses, the free-electron density can be modified, leading to the change of localized transient material properties, such as the physical properties (that is, reflectivity), so that the laser field was reshaped, then the free electron density distributions (Figure 9k–9n) and absorbed laser intensity distributions (Figure 9o–9r) can be controlled, contributing to the enhancement of the photon absorption efficiency and result in the modification improvement in the irradiated zone. We also conducted micro-Raman spectroscopy to characterize the internal structure of the sample. As Figure 9s–9u shows, by optimizing the pulse delays between the two sub-pulses, we can adjust the chemical properties (such as the Si-O bond structure) in irradiated material, which results in a higher number of 3- and 4-membered ring structures in double pulses modified regions than that in single pulse modified regions. These results lead to the increases in the reactivity of the oxygen atoms, which contributes to a higher etching rate induced by femtosecond laser double-pulse train. In short, by varying the pulse delay of pulse trains, we can control the localized transient free electron dynamics, including bound electron ionization, free-electron density, temperature and excited state distribution, and further modify localized transient material properties, such as physical and chemical properties, which in turn improve the manufacturing efficiency. Overall, femtosecond laser temporal pulse shaping fabrication that is based on EDC represents a preliminary attempt to control the chemical reaction.

#### *Modulation of femtosecond laser-induced periodic surface structures based on EDC*

On the basis of the aforementioned theory on EDC, we demonstrated that femtosecond laser-induced periodic surface structures (LIPSS, also referred as ripples) can be deliberately modulated by controlling the electron density and its distribution via designed femtosecond laser pulse trains. LIPSS have been studied extensively in various materials, including semiconductors^142, 143, 144^, metals^145^ and dielectrics^110, 146, 147^, because of their promising applications^124, 148, 149, 150, 151, 152, 153, 154^. The periodicity, orientation and structure are the typical parameters in the study of ripples. According to its periodicity, LIPSS can be divided into low spatial frequency LIPSS (LSFL) and high spatial frequency LIPSS (HSFL). It is now widely accepted that the excitation and propagation of surface plasmon polaritons (SPPs) plays a crucial role in LSFL formation^155, 156, 157^. The formation of LSFL is affected by the initial laser-SPPs interference and the subsequent grating-assisted SPPs-laser coupling effect^158, 159^. Up to now, the formation mechanism of HSFL is still under investigation. Recently, Wang *et al*^160^ demonstrated that structure evolution of LSFL and HSFL is highly dependent on the localized effective laser fluence, which determines the instantaneous optical permittivity by the laser-excited electrons creating an active plasma layer. In general, the formation mechanisms include self-organization^147, 161^, second harmonic generation (SHG)^162, 163, 164^ third harmonic generation (THG)^165^, excitation of SPPs^166^, split^167^, Coulomb explosion^168^ and cavitation instability^169^ and so on. When a femtosecond laser irradiates the surface of dielectric/semiconductor materials, free electrons can be generated, leading to the formation of electron-hole plasma (surface plasma) with time scales shorter than the electron-phonon relaxation time. The localized transient free electron density is rapidly increased through linear and nonlinear (multiphoton and avalanche) ionization, leading to the material transforming from a dielectric/semiconducting state into a metallic state^159, 170, 171, 172^. Subsequently, at the interface between the metallic state surface and air, SPs can be excited by the coupling between the surface electrons of the irradiated sample and the incident field when the real part of the dielectric function is less than −1 (Ref. 173). The SPs are characterized by surface electromagnetic waves so that the coupling field is a superposition of the incident field and the SP field. When the free electron density reaches the critical density (∼1.74 × 10^21^ cm^−3^ for the wavelength of 800 nm), the SPs can be resonantly excited^174^. The SPPs excitation and resonance can reshape the laser intensity distribution in the material and affect the subsequent linear/nonlinear ionization process. Thus, the transient free electron density and its distribution is the key factor that affects SP excitation and properties, and ultimately, the corresponding ripple formation^110, 147, 173^. Meanwhile, the increased localized electron density can be further affected by trapping, diffusion and recombination^175^ with a time scale of several hundred femtoseconds. Therefore, the time delay within the picosecond timescale is proposed to control the electron dynamics to modulate the electron density and distribution, thus to modulate the resulted ripple structures.

**Figure 10:**
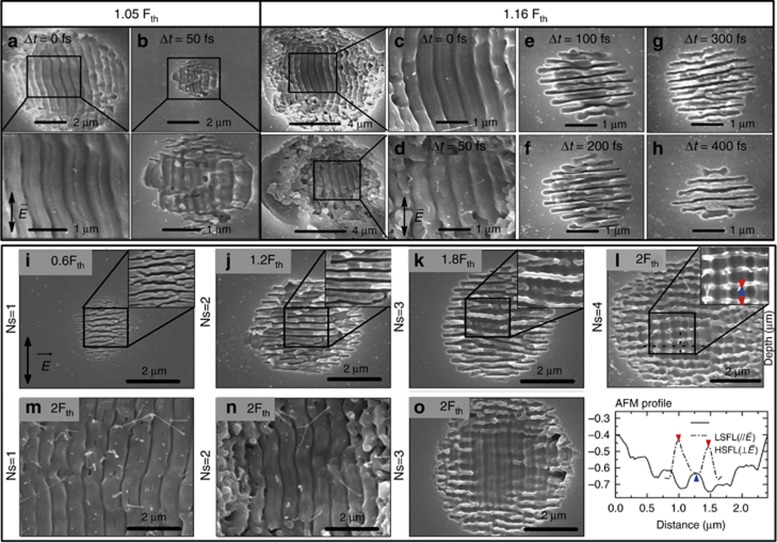
Control of period, orientation and topology of the LIPSS on the surface of fused silica via symmetrically shaped femtosecond pulses. (**a**–**h**) LSFL changes to HSFL with different orientation under certain pulse fluences and pulse delays on fused silica^122^. (**i**–**o**) SEM images of representative HSFL (**i**–**k**), LSFL (**m**–**o**) and double-grating structure (**l**) on fused silica for single-pulse trains (Ns=1), double-pulse trains (Ns=2), triple-pulse trains (Ns=3) and quadruple-pulse trains (Ns=4), respectively^123^. Reproduced from Ref. 122 (with the permission of OSA) and Ref. 123 (with the permission of OSA).

Optimal EDC using suitably shaped temporal pulse trains thus gives the possibility to modulate the LIPSS artificially, offering extended flexibility in material processing. Studies show that for femtosecond (fs) laser pulse train processing of materials, the pulse delay between sub-pulses strongly impacts the formation of nanostructures^7, 176, 177, 178, 179^, especially the morphology of LIPSS^176, 177, 178^. Here, we performed relevant experiments as examples on the surface of dielectrics to illustrate the aforementioned mechanisms^122, 123^. Fused silica was used as a dielectric material in a case study on the control of the LIPSS period, area and orientation. For conventional femtosecond laser irradiation, only LSFL with an orientation parallel to the laser polarization and HSFL with an orientation perpendicular to the laser polarization were obtained on fused silica depending on the laser fluences (*F*) or pulse number (*N*). Nevertheless, compared with the conventional situation, both types of ripples with controllable periods, areas and orientations, especially the HSFL with an orientation parallel to the laser polarization, were obtained by changing the pulse delay (Δ*t*) and pulse fluence (Figure 10 upper panel). Thus, three types of LIPSS under specific conditions can be obtained. (1) LSFL with orientation parallel to the laser polarization direction; (2) HSFL with orientation parallel to the laser polarization direction with low pulse fluence; and (3) HSFL with orientation perpendicular to the laser polarization direction at higher pulse fluence. The experimental results indicate that: (1) at lower pulse fluences, a transition from LSFL to HSFL occurred at a pulse delay of 50 fs with a decrease in area (Figure 10a and 10b,); (2) whereas, at higher fluences, LSFL were replaced by another type of HSFL with an orientation perpendicular to the laser polarization at Δ*t*>100 fs (Figure 10c–10h). The average periods of LSFL and HSFL were 560±8 nm and 255±30 nm, respectively.

During processing, the second sub-pulse of the doublepulse train significantly affects the free electron density and distribution generated by the first sub-pulse, thereby influencing the mechanism of LIPSS formation and the surface morphology. Consequently, by controlling the pulse delay and pulse fluence, we can control excited electron production, distribution, motivation and the interaction between surface plasmon (SP) and the incident laser, then to control the periodicity, orientation and morphology of the LIPSS.

1. LSFL obtained here at a low pulse fluence were oriented parallel to the laser polarization, which cannot be explained by SPs excitation^147, 180^. According to calculations based on the Sipe-Drude model^180^ and pump-probe results^53^, femtosecond energy deposition by the first/previous pulses can occur at specific (LSFL) spatial frequencies, reshaping the electron density distribution along the polarization direction, and then determines the formation of LSFL with an orientation parallel to the polarization direction. Meanwhile, thermal effects also play a critical role in subsequent material removals at the fluences for LSFL generation^181, 182, 183^;
2. The periods of LSFL and HSFL (with orientation parallel to the polarization direction) were close to the fundamental (*λ*/n=551 nm) and second-harmonic (*λ*/2n=275 nm) wavelengths in fused silica with lower pulse fluence. Thus, SHG plays a key role in HSFL formation with an orientation parallel to the polarization direction^155, 156^ at a lower pulse fluence. SHG is a result of electron recombination, which is determined by the electron density. By controlling the pulse delay, the electron occupation is adjusted^110^, leading to the manipulation of electron density, thus facilitating SHG and resulting in a 50% cut in LIPSS periods;
3. The strong decrease in the ripple area was due to the electron decay. The free electrons in the conduction band excited by the first sub-pulse relaxed and returned to the valence band during the time interval between the two pulses, in terms of diffusion and recombination^184, 185, 186^, leading to the reduced electron density; therefore, the energy coupling by the second sub-pulse to the excited material decreased, leading to the decay of ablation, which resulted in the reduced rippled area;
4. The transition of HSFL at a higher pulse fluence was mainly attributed to the periodic plasma enhancement of the incident laser field, which is related to the excited free electron density. When the pulse delay was low (<100 fs), the effects of induced SPs on the incident laser were insufficient due to the low absorbed intensity. With pulse delays of 100–500 fs, however, SPs excitation can easily be achieved at the initial stage of the second sub-pulse due to the accumulation of the first sub-pulse^110^ (see modeling section for details). The interaction between SPPs and the incident laser field resulted in the periodic modulated intensity enhancement at the surface. The incubation effects with multiple bursts irradiation led to the evolution of the local intensity distribution along the electric field direction^187^, resulting in HSFL formation at a specific period with an orientation perpendicular to the electric field direction.

More complicated morphology control of LIPSS on fused silica can be obtained by EDC via shaping the conventional femtosecond laser pulses into symmetrical triple- and quadruple-pulse sequences, especially, double-grating structures and an HSFL period as small as 190 nm were obtained (see Figure 10i–10o)^123^. In addition, the geometric morphology modulation on Si can also be obtained by EDC via adjusting double pulse delay^140^. Therefore, the aforementioned experimental studies demonstrate that by designing a femtosecond laser pulse train, the electron dynamics can be controlled, that is, electron density, distribution, thus to control the coupling between the SPP and incident laser, and finally guide the material response (LIPSS morphology) towards user-designed directions with various morphologies (for example, periods, orientations, distributions and geometric morphologies).

#### *Detection sensitivity improvement of SERS based on EDC*

**Figure 11:**
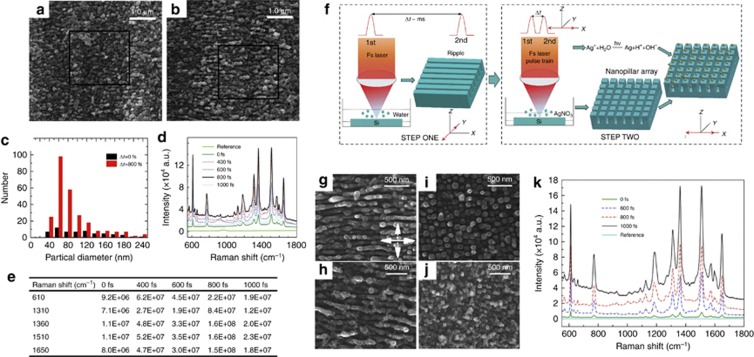
(**a**–**e**) One-step method fabrication of controllable SERS substrates^125^. (**a** and **b**) SEM images of the silicon irradiated at pulse delays of 0 fs, and 800 fs, respectively. (**c**) Size distribution of silver nanoparticles in **a** (black), and **b** (red). (**d**) SERS signals of R6G molecules on the as-prepared substrates at various pulse delay. (**e**) Enhancement factors with different pulse delays. (**f**–**k**) Two-step method fabrication of controllable SERS substrate^126^. **f** Schematic diagram of the SERRS substrate fabrication process. (**g**–**j**) SEM images of silicon substrates irradiated at pulse delays of **g** 0 fs, and **i** 1000 fs in deionized water, (**h**) 0 fs and (**j**) 1000 fs in 10-mM silver nitrate solution. (**k**) SERS spectrum of substrates fabricated at different pulse delays. Reproduced from Ref. 125 (with the permission of OSA), and Ref. 126 (with the permission of OSA).

By designing femtosecond laser pulse trains on the basis of EDC, we can control the localized transient electron density and its distribution to modulate the properties of SPs, then promote the energy transfer to materials and control the surface structures and photochemical reduction process; thus, high detection sensitivity SERS substrates can be achieved. SERS has been recognized as the most promising trace analyte detection method for rapid and accurate label-free analysis of chemical and biological species because of its high sensitivity and fingerprint-identification features^188, 189^. Previous studies have demonstrated that surface morphologies (for example, ripples, nanoparticles and nanopillars)^190, 191^ play key roles in SERS enhancement in which the enhanced electromagnetic field on surface nanostructures induced by the localized surface plasmons resonance (LSPR) effect dominated^191, 192, 193^. Consequently, tailoring the surface structures into different morphologies and sizes, in this aspect, is significant for tuning LSPR features to further improve the sensitivity and push SERS devices into practical applications. As shown in Figure 11d, the SERS intensity gradually reached a maximum when the pulse delay was increased from 0 to 800 fs, and then decreased when the pulse delay was further increased to 1000 fs^125^. Although the small changes (that is, the modulation of pulse delays) occurred in the incident pulses, the obtained surface structures significantly improved the signal intensity (Figure 11e). Compared with the conventional femtosecond laser ablation (Δ*t*=0 fs), the designed pulse train could reduce more silver nanoparticles (Figure 11a and 11b) and lead to a more uniform distribution of the nanoparticles deposited on the subwavelength ripples (Figure 11c). Consequently, the SERS sensitivity was improved.

To further confirm that we controlled the properties of SPs, a two-step experiment was conducted using a designed pulse train, as schematically shown in Figure 11f^126^. The laser polarization and pulse delay were synergistically controlled. Compared with the ripples formed by conventional femtosecond laser pulses (Figure 11g and 11h), regular nanopillar arrays were generated by a double pulse train (i.e., Δ*t*>0 fs) with the polarization direction rotated by 90°, as shown in Figure 11i and 11j. In the case of conventional femtosecond laser ablation (Δ*t*=0), SP induced by the previous pulse of the subwavelength ripples would fast damp to its original state before the subsequent pulse arrived because of the long-time interval (millisecond scale), leading to the formation of large and non-uniformly distributed silver nanoparticles on conventional grating-like ripples. By contrast, because the pulse delay of a pulse train is shorter than the damping time of SP, the properties of SPs would be significantly controlled to change the energy transfer efficiency, which could contribute to the formation of abundant small and uniformly distributed silver nanoparticles on the nanopillar arrays. In addition, the larger enhancement of the incident laser electric field on nanopillar arrays lead to generation of much more silver nanoparticles, which resulted in a lager SERS signals (maximum enhancement factor up to 2.2 × 10^8^). Overall, by designing a pulse train on the basis of EDC, the electron density and its distribution induced by laser irradiation can be controlled to modulate the properties of SPs, resulting in a more effective energy transfer and changes in the resulting structures. These findings provide new insights regarding tuning LSPR features for related applications.

#### *Surface chemical reduction activity of MoS_2_ controlled by temporal pulse shaping based on EDC*

**Figure 12:**
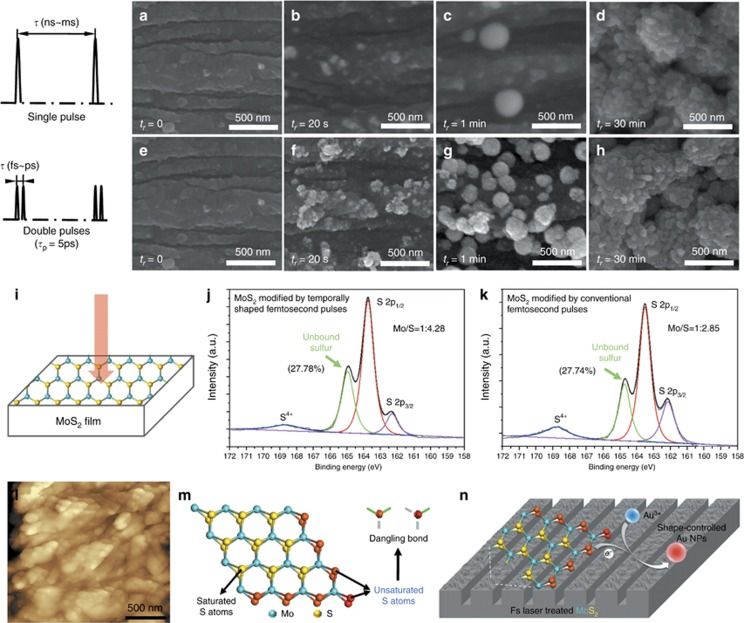
Morphology evolution of gold NPs reduced on MoS_2_ surfaces irradiated by femtosecond (**a**–**d**) single and (**e**–**h**) double pulses, at different stages of the reduction process, where tr represents the chemical reduction time, the pulse delay is 5 ps. (**i**) Schematic diagram of the manufacturing and Mo-S bond structure. XPS S 2p spectra of modified regions irradiated by femtosecond laser (**j**) double and (**k**) single pulses on MoS_2_, where the percentage value represents the content of unbound sulfur and the atomic ratio represents the relative atomic concentration ratio of Mo and S atoms. (**l**) AFM image and (**m**) atomic scale schematic of the laser-broken micro/nano MoS_2_ debris. (**n**) Mechanism of chemical reduction of gold cations on laser-treated MoS_2_ (Ref. 127). Reproduced from Ref. 127 (with the permission of ACS).

By designing femtosecond pulse trains on the basis of EDC, bound electrons can be ionized, chemical bonds between atoms can be interrupted, atoms can be selectively removed and material properties can be modified so that gold cations can be spontaneously reduced on MoS_2_ surface^127^, as shown in Figure 12. Compared with the conventional femtosecond laser pulses, the chemical reduction rate of gold cations on MoS_2_ surface modified by femtosecond double-pulse trains was significantly enhanced (Figure 12a–12h). We conducted X-ray photoelectron spectroscopy (XPS) and atomic force microscope (AFM) to characterize and analyze the element valence, atomic ratio and morphology of the sample, as shown in Figure 12i–12m. When material was irradiated by the femtosecond laser pulses, electrons were excited from bonding to anti-bonding states (bound electrons were ionized), hence abundant chemical bonds between atoms (such as Mo-S bonds) were instantaneously weaken or even broken entirely, resulting in an integral increase in the binding energy of S atoms and the appearance of unbound sulfur (unsaturated S atoms with dangling bonds). Meanwhile, abundant Mo atoms were selectively removed, and the surface lattice structure of the material was non-perfect and broken into a large number of micro/nano debris terminating with unsaturated S atoms (edge active sites). These unsaturated-terminal S atoms induced on MoS_2_ can reduce gold cations to gold atoms (Figure 12n). Compared with conventional femtosecond laser pulses, more Mo atoms were selectively removed and more unbound sulfur was formed, which might result from the significantly enhanced ionization of bound electrons by femtosecond double-pulse trains (first, bound electrons were ionized by the first sub-pulse and the electron-hole pairs separated; second, the photoexcited electron-hole pairs recombined, which began within 500 fs and would last for more than a hundred picoseconds; last, the second sub-pulse could further ionize the recombining electron-hole pairs, which might further facilitate the photochemical bond breaking)^38, 194, 195^ and inevitably resulted in stronger chemical reduction activity and higher chemical reduction rate on laser-treated MoS_2_. In conclusion, by designing pulse trains based on EDC, the bound electrons ionized by laser irradiation can be controlled to modulate chemical bond cleavage and increase chemical reduction ability, which can reduce gold cations to obtain metal-MoS_2_ hybrids for relevant applications.

#### *High-quality fabrication by temporal pulse shaping based on EDC*

**Figure 13:**
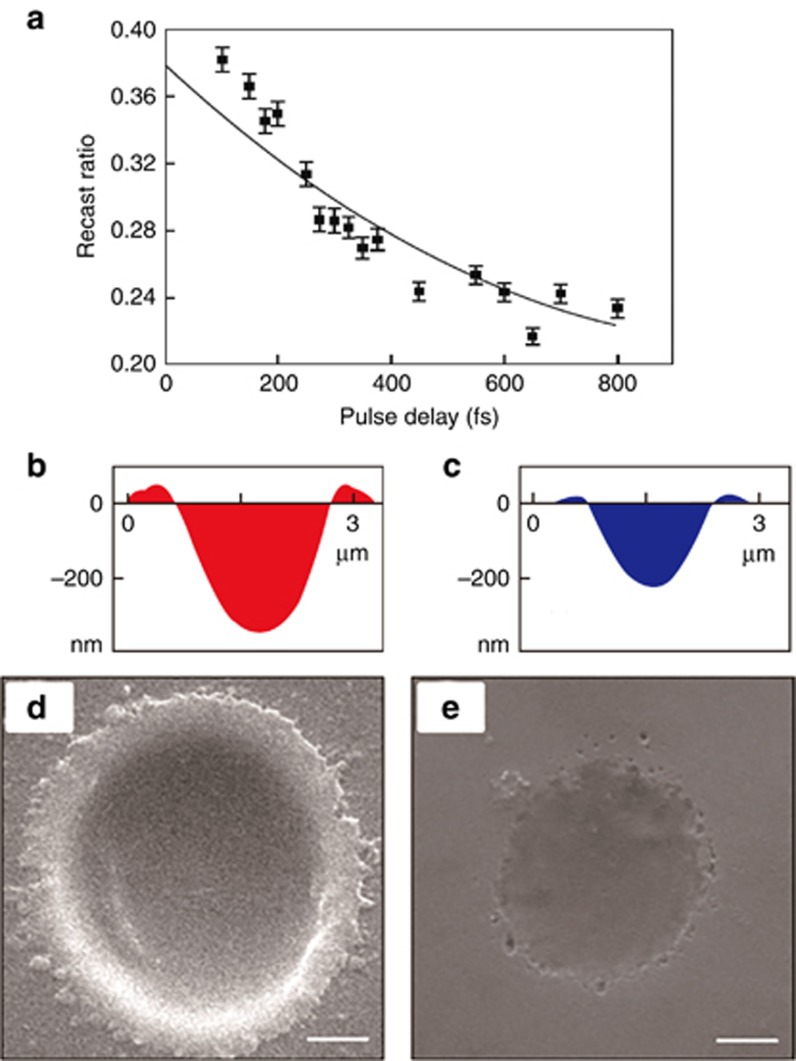
(**a**) Dependence between the recast ratio (recast area/ablation area) and pulse delay on fused silica fabrication using femtosecond laser pulse trains consisting of two identical sub-pulses with an identical total fluence. AFM profiles of the structures of the fused silica fabrication using (**b**) a conventional single pulse and (**c**) femtosecond laser pulse train with a pulse delay of 300 fs. SEM images of the structures on fused silica fabrication using a femtosecond laser pulse train with different energy ratios between the two sub-pulses: (**d**) 1:1 and (**e**) 2:1. The femtosecond laser with wavelength of 800 nm, duration of 35 fs and repetition rate up to 1 KHz. The total fluence of the pulse trains in all experiments is 5 J cm^−2^; the scale bar in **d** and **e** is 500 nm. Reproduced from Ref. 128 (with the permission of OSA).

By designing femtosecond laser pulse trains on the basis of EDC, the phase change mechanism can be controlled to achieve high-quality fabrication^128^. As Figure 13a shows, the recast ratio (recast area/ablation area) of the ablation structures on the fused silica decreased as the pulse delay increased within a femtosecond laser double-pulse train. Compared with a conventional single pulse, the recast height surrounding the ablation spot decreased by 60% when fabrication was performed using a femtosecond laser pulse train (see Figure 13b and 13c, for atomic force microscope (AFM) profiles). In this case, the total fluence of the pulse train was 5 J cm^−2^ (greater than the ablation threshold of fused silica). The conventional femtosecond laser single pulse induced an electron density much higher than the critical density and a higher Coulomb barrier, therefore leading to significant electron screening effects. Consequently, the accumulation of positive charge during ablation was reduced, thus the electric field was weakened so that the effectiveness of the nonthermal processes (Coulomb repulsion and/or electrostatic ablation) was reduced. The thermal phase change including melting (various phase change mechanisms coexisted during the process) dominated the ablation process, which resulted in inferior fabrication quality. While in the case of femtosecond laser pulse train with the identical total fluence, a single pulse was split into two sub-pulses with the identical fluence lower than the ablation threshold of fused silica. Therefore, through optimizing the pulse delay between the two sub-pulses, the laser-induced electron density can be controlled to be slightly higher than the critical density. Subsequently, the nonthermal phase-change mechanisms mainly dominate the fabrication process, contributing to less recast and high fabrication quality. Furthermore, by adjusting of the energy ratio between the two sub-pulses, the ionization processes were altered to change the pulse energy absorption. Consequently, the free electron distribution can be adjusted to make the phase change process a nonthermal one. Therefore, we achieved much higher fabrication quality and more controllable structures (Figure 13d and 13e). Overall, through femtosecond laser temporal pulse shaping, the localized electron dynamics of the materials can be controlled, thus the phase-change mechanisms can be adjusted to be dominated through nonthermal phase change process so that higher fabrication quality and more controllable structures can be achieved.

### Femtosecond laser spatial pulse shaping

**Figure 14:**
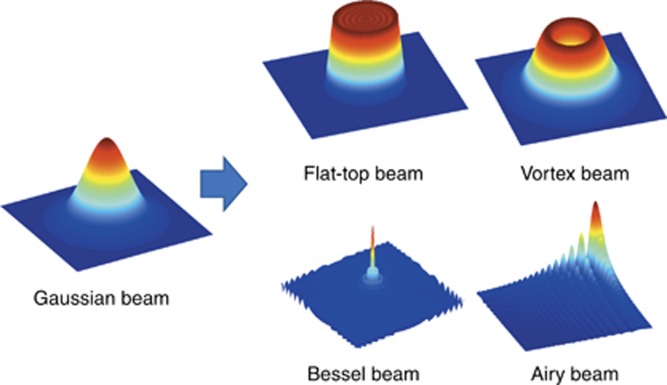
Shaping conventional Gaussian beam into different beam types.

Spatial laser shaping is another key aspect of EDC. The spatial states of electron dynamics are closely related to the spatial distribution of laser energy. Conventionally, most commercial laser systems provide a fundamental Gaussian intensity profile, which has a short Rayleigh length, small beam waist of focusing spot, homogeneous phase/polarization state and limited numbers of laser spots. These characteristics of Gaussian laser cannot satisfy the increasingly higher demands for flexibility, precision and efficiency in high-end laser fabrication. Thus, it is essential to spatially shape the laser pulses to control the spatial electron density/temperature distribution. By spatial pulse shaping, the Gaussian beam can be converted into different beam types, such as flat top beam, vortex beam and Bessel beam, Airy beam and so on (Figure 14) Some unique characteristics of these beam types can lead to special functionality for laser fabrication, which has a large advantage over conventional Gaussian beams.

#### *Spatial pulse shaping experimental setup*

**Figure 15:**
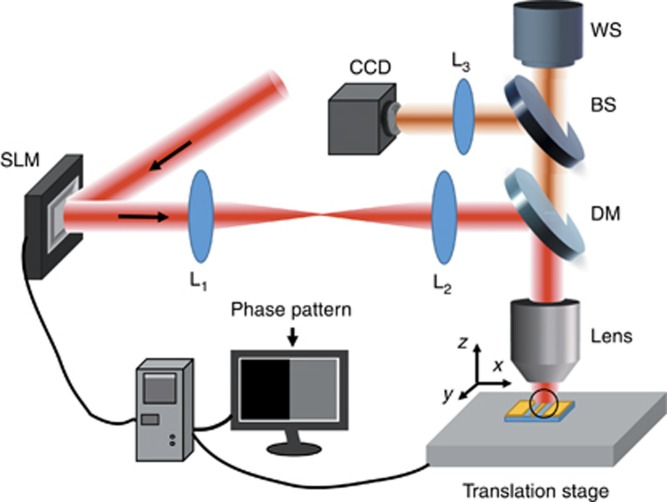
Schematic diagram of the experimental setup. WS: white light source; BS: beam splitter; DM: dichroic mirror; L1, L2: two convex lenses consisting of a 4f relay system; L3: convex lens; Inset: section of the samples and the focusing laser in the *yz* plane^129^. Reproduced from Ref. 129 (with the permission of Wiley).

The experimental setup is shown in Figure 15. An amplified Ti: sapphire laser system (Spitfire Ace-35F Spectra Physics Inc.) provides a fundamental Gaussian mode with a central wavelength of 800 nm and a pulse duration of 35 fs. Phase patterns were generated using the liquid crystal on a silicon spatial light modulator (SLM, Holoeye Pluto). The size of the liquid crystal screen was 15.36 × 8.64 mm. The modulated laser beam passes through a 4f relay system, which consisted of two plano-convex lenses (L1, L2). The distance between the SLM and lens L1 was equal to the L1 focal length. The distance between the two lenses was the sum of their focal lengths. The laser beam can then be transmitted to the focal plane of the second lens L2 without any distortion. The focal length was 100 mm (L1) and 150 mm (L2), respectively. The samples were mounted on a nanometer-precision stage (Newport, NPXYZ100) has a resolution of 0.2 nm in the *X*, *Y* and *Z* planes. The fabrication process was monitored using a CCD camera and white light source (WS).

#### *High precision nanowire fabrication by spatial pulse shaping based on EDC*

Diffraction is a universal phenomenon in wave optics, which greatly limits the resolution of laser fabrication to half of the wavelength level. One of the greatest challenges in laser micro/nano fabrication is ‘overcoming’ the diffraction limit. Several methods have been developed to solve this problem using different mechanisms, such as near-field fabrication^196, 197^, two/multi-photon polymerizations^198^ and plasmonics-based fabrications^199^. However, these methods all have different disadvantages, including the low efficiency^196, 197^, complex procedures^196, 197^, weak flexibility^199^. and limited types of applicable materials^198^. A novel simple, repeatable, mask-free, high-throughput, broad-applicability and high-flexibility method is highly desired.

**Figure 16:**
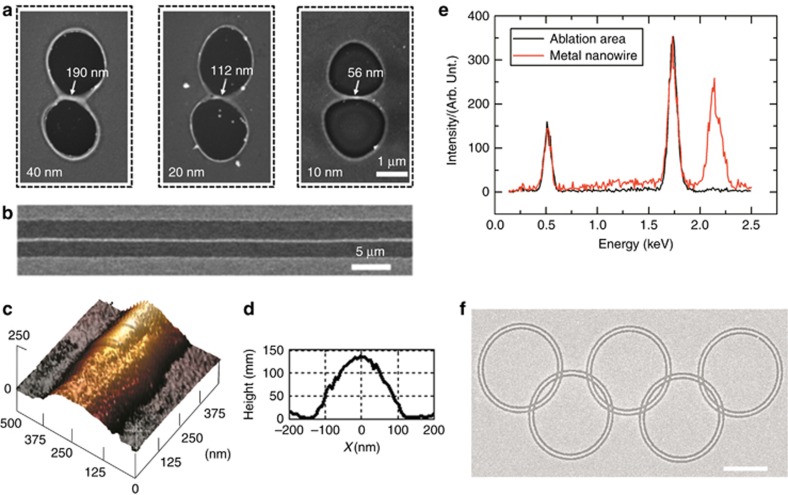
Fabrication of nanowire by spatial pulse shaping. (**a**) Single spot fabricated by the shaped beam. (**b**) Scanning electron microscope (SEM) images of nanowire. (**c** and **d**) AFM images of the nanowire and its cross section. (**e**) EDXS measurements of the metal nanowire and the ablation area. (**f**) Five-ring patterns fabricated by the proposed methods^129^. Reproduced from Ref. 129 (with the permission of Wiley).

By using a spatially modulated femtosecond beam based on spatial EDC, we achieved high-resolution nanowire patterning that breaks the light diffraction limit^129^. The initial beam had a Gaussian intensity profile with an even phase wave front. The phase of the initial beam was modulated using a liquid crystal on silicon spatial light modulator. After modulation, a relative phase difference was created between two equal parts of the incident beam. Subsequently, the modulated beam was focused by an objective lens, forming a dual-peak focusing spot with an intensity valley in the center because of a diffraction effect. The spatially modulated beam was used to pattern a gold thin film, which was deposited on a silica substrate by electron beam evaporator. The central thin film was preserved due to the intensity valley thus a nanowire was formed in the beam center. Arbitrary nanowire can be generated on the substrate by dynamically adjusting the orientation of the intensity valley. A minimum nanowire width of approximately 56 nm (∼1/14 of the laser wavelength) can be achieved (Figure 16a–16d). The high resolution is achieved by combining the ultrashort nature of the femtosecond and the low thermal conductivity of the thin film. We tested the amount of Au residue using an electron diffraction X-ray spectrum (EDXS) experiment (Thermo Scientific, MA, USA; Figure 16e). By changing the direction of the phase pattern loaded on the SLM, the center nanowire changed its orientation accordingly. By dynamically adjusting the direction of the phase pattern to tangent of the scanning route, arbitrary curves can be fabricated. Figure 16f shows Olympic rings fabricated using this method, which evidence its effective curve patterning ability.

**Figure 17:**
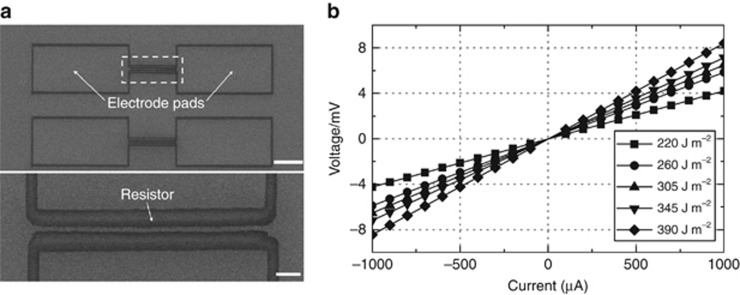
Testing of the resistivity of the nanowire. (**a**) The SEM images of the nanowire and the electrode pads. (**b**) The volt-ampere characteristics of the nanowires. Reproduced from Ref. 129 (with the permission of Wiley).

To investigate the electronic characteristics of the nanowire, the volt-ampere characteristics curve of the nanowire fabricated by different energy pulses was measured. (Figure 17) No inner nanopores and particle intervals are generated inside the nanowire, thus endowing the nanowire with favorable electronic characteristics: the conductivity of the nanowires was as high as 1.2 × 10^7^ S m^−1^, and the maximum current density was up to 1.66 × 10^8^ A m^−2^. This approach offers a simple, robust alternative for high-quality nanowire fabrication as a complementary method to conventional lithography methods.

## Multiscale measurement of electron dynamics during femtosecond laser-material interactions

**Figure 18:**
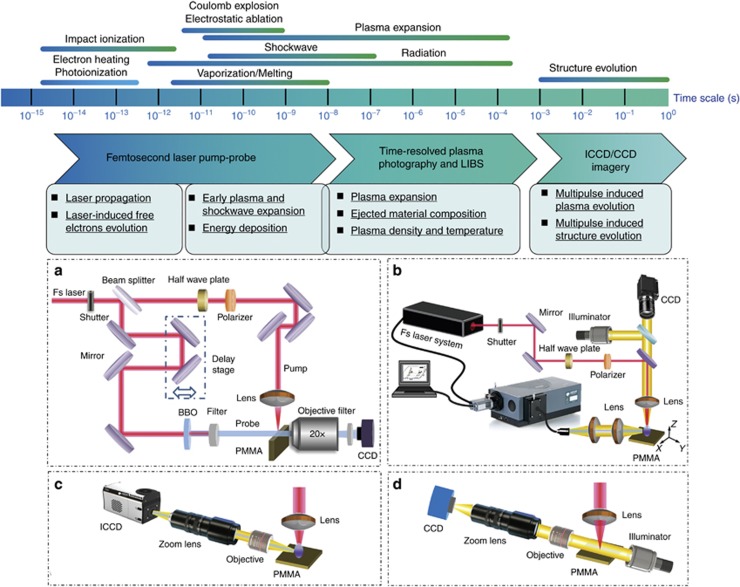
Schematic of multiscale measurement of femtosecond laser drilling, including laser propagation and laser-induced material excitation, plasma and shockwave evolution and hole formation and so on. (**a**) Pump-probe shadowgraph imaging technique. (**b**) Laser-induced breakdown spectroscopy (LIBS). (**c**) Time-resolved plasma photography with gated intensified charge-coupled device (ICCD). (**d**) Industrial continuous imagery.

To comprehensively understand the electron dynamics during femtosecond laser micro/nano fabrications, a multiscale measurement system was developed to monitor the spatiotemporal electron dynamics of laser-material interactions. This system integrates the widely applied ultrafast pump-probe microscopy^198, 199^, time-resolved plasma photography with a gated intensified charge-coupled device (ICCD)^200, 201^, LIBS^202, 203, 204^ and industrial continuous imagery^205^(Figure 18). These techniques have different characteristic time resolutions, ranging from femtoseconds to seconds. By virtue of the multiscale ability, the electron dynamics of femtosecond laser micro/nano fabrications can be revealed at different scales.

**Figure 19:**
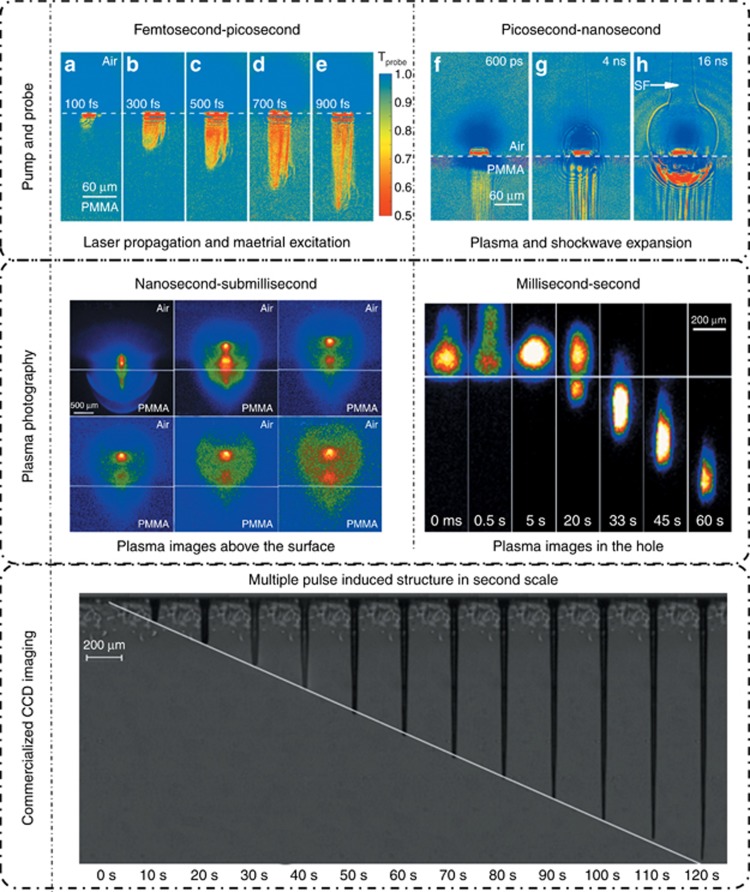
Multiscale measurement results of deep-hole drilling process in PMMA. Multiscale measurement results of deep-hole drilling process in PMMA with 100 μJ pulse energy focused by plano-convex lens (*f*=100 mm). The dynamics include femtosecond-picosecond electron excitation, picosecond–nanosecond plasma and shockwave evolution and multiple pulse-induced structure in second scale.

As a case study, the deep-hole drilling process in poly (methyl methacrylate) (PMMA) was investigated using the multiscale measurement system, as shown in Figure 19. In the femtosecond to picosecond time scale, focused laser propagation in the material, as well as free electron generation, diffusion and recombination, was detected through pump-probe shadowgraphy (Supplementary Movie 2,Supplementary Information). Nonlinear phenomena were observed at a high pulse energy as a result of the strong self-focusing effect of the intense femtosecond laser. In the picosecond to nanosecond time scale, the early plasma expansion and following shockwave evolution in the atmosphere can also be studied through pump-probe shadowgraphy (Supplementary Movie 3, Supplementary Information). Shockwave expansion properties were investigated on the basis of the Sedov-Taylor solution, revealing the change of environment and its effect on laser ablation. For larger time scales, time-resolved plasma photography with the gated ICCD and LIBS were employed to collect the plasma expansion morphology (Supplementary Movie 4, Supplementary Information) and plasma emission spectroscopy, respectively. These results not only provided the expansion dynamics of plasma but also the plasma intrinsic information, including the material composition, plasma density and temperature. In the millisecond to second time scale, the plasma evolution induced by multiple pulses was studied using time-resolved plasma photography, revealing the effect of the prior structure on the plasma intensity and distribution (Supplementary Movie 5, Supplementary Information). Furthermore, microhole formation was studied using industrial continuous imagery (Supplementary Movie 6, Supplementary Information), providing information concerning the evolution of the depth, diameter and quality of the high-aspect-ratio microhole.

By using the measurement system, we determined the nanosecond-scale electron temperature and density evolution under double pulse irradiation. Large differences in plasma plume ejection of PMMA and fused silica were demonstrated using the LIBS technique, following the different electron dynamics designed by femtosecond laser double-pulse train. By changing the pulse delay, we could modify the generation and distribution of electrons and thus effectively improve the electron density and temperature. Finally, we could improve the plasma enhancement factor and optimize the ablation accuracy through EDC.

**Figure 20:**
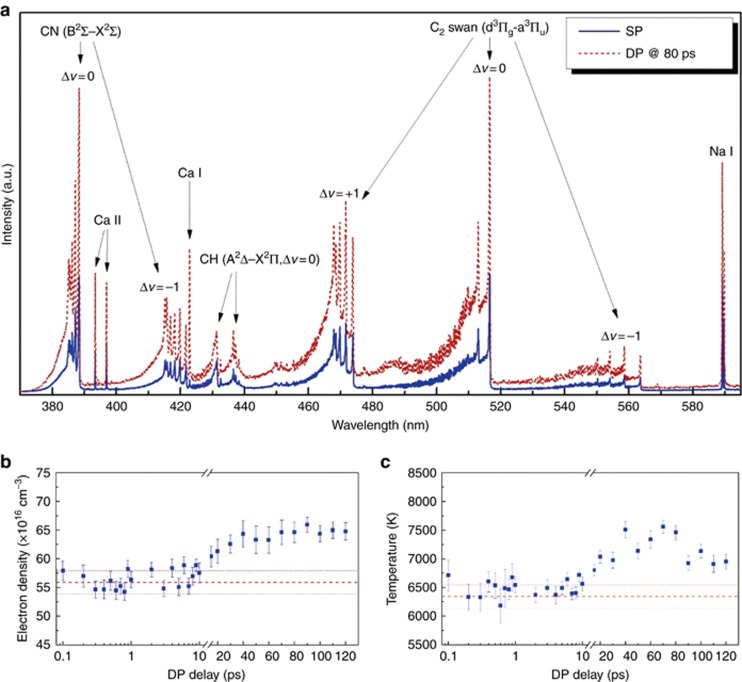
Typical spectra of PMMA plasma irradiated by a single pulse and a double pulse with the same total fluence of 7.8 J cm^−2^. Reproduced from Ref. 134 (with the permission of SPIE).

The plasma spectra of PMMA induced by single pulse and double pulse irradiation at the same laser fluence were compared to explore the LIBS enhancement mechanism. The plasma spectra of PMMA consisted of emission peaks of molecular species (CN, CH and C_2_) as well as atomic and ionic species (Ca I, Na I and Ga II)^134^. As Figure 20 shows, double-pulse-induced plasma emission signal enhancement strongly depended on the pulse delay; the signal was stronger than that of single-pulse irradiation when the pulse delay exceeded 10 ps. The maximum enhancement value was about 7 at the pulse delay of approximately 80 ps. To determine the electron dynamics difference, the plasma temperature and electron density were calculated using the Saha–Boltzmann plot and the Stark broadening method, respectively. The plasma temperature variation was correlated with the signal enhancements achieved using the double-pulse delay, implying that the plasma reheating effect of the second pulse was the main mechanism of the enhancement effect. By contrast, the maximum enhancements were different for molecules, atoms and ions, which were related to the different upper excitation energy of the emission transition and the ionization stage of the electrons^200, 201^.

**Figure 21:**
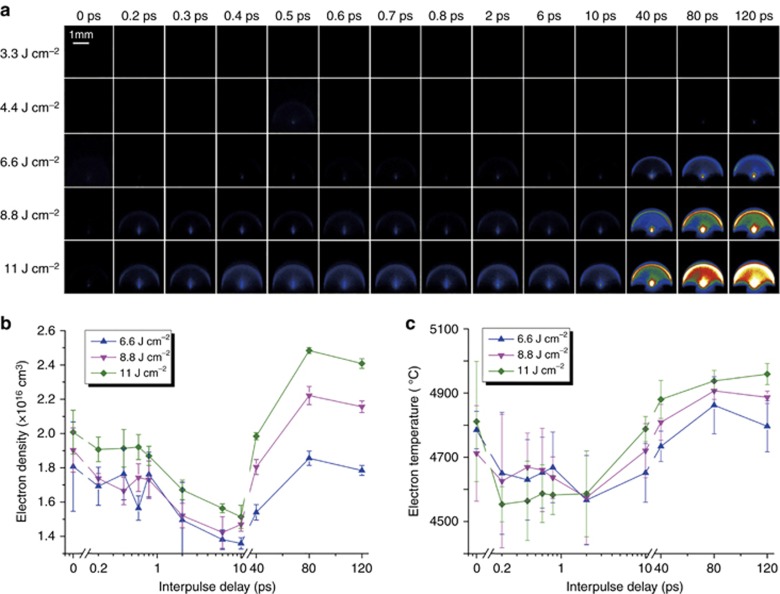
Characterizations of femtosecond laser double pulse induced plasma of fused silica (**a**) plasma images, (**b**) electron densities and (**c**) plasma temperature.

A comparison of fused silica plasma emission induced by a single pulse and that induced by a double pulse is presented in Figure 21a and 21b. The plasma emissions at the pulse delay below 10 ps were stronger than they were in the single-pulse case. This phenomenon was explained by the existence of free electrons and self-trapped excitons at this pulse delay. The free electrons and self-trapped excitons left by the first pulse increased the absorption efficiency of the second pulse. Moreover, extraordinarily high enhancement factors were observed at a pulse delay above 10 ps. The maximum enhancement factor of double-pulse irradiation was ∼35 times at a pulse delay of 120 ps for a fluence of 11 J cm^−2^. The plasma consisted of a fast component (ionized atoms and ions) and a slow plume component (partially ionized nanoparticles), with the slow part contributing little to plasma emission^203^. The ionization of the slow part by the second pulse greatly increased the plasma quantity and was demonstrated to be the main cause of the high enhancement factor when the pulse delay was larger than 10 ps. The plasma temperature was also calculated to ascertain its changes with respect to the pulse delay (Figure 21c). Hence, the electron dynamics (free electrons, self-trapped excitons or nanoparticles) left by the first pulse dominated the plasma emission intensity of the femtosecond double pulse LIBS.

## Applications of EDC in high quality and aspect-ratio microholes drilling

As discussed before, because of the significant electron-lattice nonequilibrium state in the femtosecond laser fabrication process, the laser-material interactions, including phase change and material removal, are actually determined by the initial photon–electron interactions^26, 27^. Furthermore, the electron density distribution can be manipulated by modifying the laser intensity distribution. By implementing EDC through spatial pulse shaping, forming an intense, long and uniform Bessel beam, to adjust the localized transient electron density distribution, and thus control phase change, we fabricated high quality and high aspect-ratio microholes. The technique was applied in key structure fabrication in one of the 16 Chinese National S&T Major Projects.

Microholes fabrication in the key structure faced many challenges, including the high aspect-ratio (>20:1), small diameter (<10 μm), taper-free, high quality, reduced recast/ejected materials and minimized in-cavity residues. Although various novel methods had been proposed for microholes fabrication, there were many challenges that limited their application in the key structure fabrication. By using a particle based near-field nanostructuring to overcome the optical diffraction limit, Quentin *et al*^203^ achieved nanoholes with diameters below 200 nm, but the structure was a shallow crater and the aspect-ratio was below 1:1. He *et al*^204^ reported that high aspect-ratio microholes were fabricated in fused silica with femtosecond laser transverse direct-writing followed by wet chemical etching, but the cross-sections of the microholes fabricated by this method are usually in poor shape ascribing to the asymmetric shape of the focal spot. Gottmann *et al*^205^ constructed a selective laser-induced etching system of 3D precision quartz glass components. By the combination of a three-axis system to move the glass sample and a fast 3D system to move the laser focus, the selective laser-induced etching process (LightFab 3D Printer) is suitable to produce more complex structures in a shorter time. However, the over-etching for some complex structures and the formation of cracks during the etching process limit advanced applications. In addition to the aforementioned methods, percussion drilling has attracted the most interests^204, 206, 207^. Nevertheless, the aspect-ratio of microholes with percussion drilling is usually smaller than 10:1 in air because of the saturation effect and the occurrence of the bending effect. We recently reduced the ambient pressure from approximately 10^5^ Pa (air) to ∼1 Pa (rough vacuum) to control the expansion dynamic of the ablated plasma/material^133, 208^. The aspect ratio of the microholes was significantly improved from ~40:1 (in air) to approximately 100:1 (in vacuum), and the bending effect was simultaneously eliminated. Nonetheless, conventional Gaussian beam drilling could barely achieve a much higher aspect-ratio of taper-free microholes with diameters below 10 μm.

To overcome the disadvantages of the traditional Gaussian beam drilling, we proposed a new processing method for high aspect-ratio and high-quality microdrilling by optimizing the localized transient electron density distribution in plasma in the focal spot through spatial pulse shaping^132^. Taper-free microholes with a diameter of approximately 1.6 μm and an aspect ratio of up to 330:1 using a single spatial shaping pulse (Bessel beam). The aspect ratio of these fabricated microholes was 52 times larger than those formed by using a Gaussian beam in similar focusing conditions. The formation of these high aspect-ratio microholes is attributed to the intense, long and uniform transient localized electron density distribution, which is adjusted on the basis of the unique intensity distribution and propagation stability of the Bessel beam through spatial pulse shaping.

**Figure 22:**
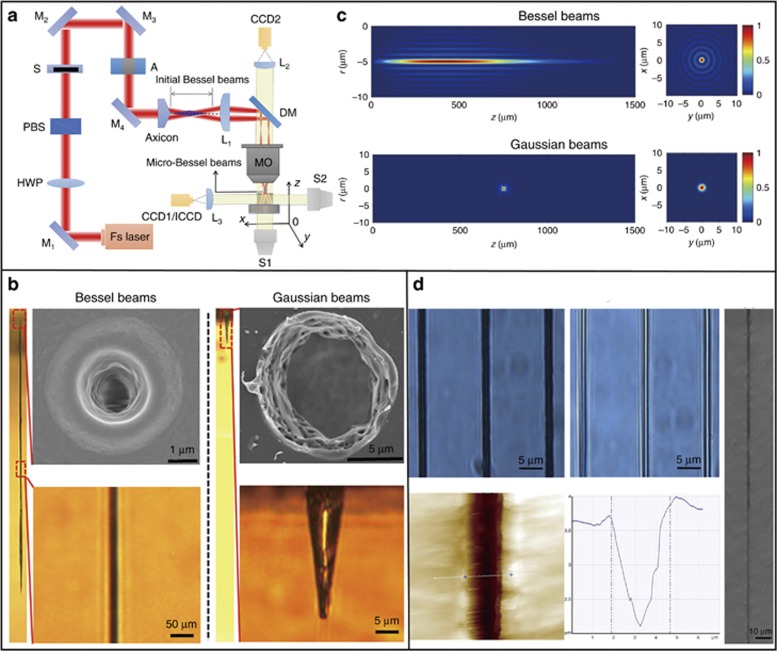
(**a**) Schematic of the spatial shaping femtosecond laser pulses microdrilling setups; (**b**) Morphology images of microholes drilled with a single pulse Bessel beam and Gaussian beam, respectively; (**c**) Intensity distribution simulations of the Bessel beam and Gaussian beam; (**d**) The hollowness characterization of microholes drilled by single-pulse Bessel beam. Reproduced from Ref. 132 (with the permission of Springer).

In our experiments, we normally used an axicon (Edmund Inc., Barrington, NJ, USA, base angle *α*=2°, refractive index=1.45) to transform a Gaussian beam into a Bessel beam for high aspect-ratio, high quality and high efficiency microdrilling. This is a convenient and effective method for spatial pulse shaping. Figure 22a shows the schematic of the experimental setup used for femtosecond laser microhole drilling^132^. Subsequently, we performed the experiments to prove that high aspect-ratio and high quality microholes can be obtained by using a single spatial shaping pulse. As Figure 22b shows, a microhole with a mean diameter of 1.5 μm and a depth of approximately 523 μm can be drilled using a single-pulse Bessel beam at a pulse energy (E) of 20 μJ in PMMA. Under identical processing conditions, the diameter and depth of a microhole fabricated using the Gaussian beam were 7.2 and 41 μm, respectively. Subsequently, to further demonstrate the hollowness of the microholes through single spatial shaping pulse drilling, the two different methods were mainly adopted, namely the liquid infiltration method and the cross-section profile test, as shown in Figure 22d. The microholes drilled using this method were small in diameter and exhibited a high aspect-ratio, a taper-free sidewall, a highly circular entrance and fewer surface-ejected materials.

Moreover, we performed a series of theoretical investigations and simulations on the spatial intensity distribution of Bessel beams and Gaussian beams to more thoroughly understand the physical mechanisms of pulse microdrilling with spatial shaping. On the basis of the theory of spatial pulse shaping using the axicon, we respectively simulated the spatial intensity distribution of single-pulse, micro-Bessel beam and a focused Gaussian beam in the longitudinal and transverse plane, as shown in Figure 22c. The micro-Bessel beam exhibited a large focal depth (*Z*_max_=597 μm) in comparison with the focused Gaussian beam (Raleigh range *R*=18.5 μm). According to the simulation results, the single-pulse Bessel beam exhibited an intense, long, consistent and stable intensity distribution without any nonlinear beam distortion along the propagation direction. These unique properties of the spatial shaping pulse allow for uniform energy deposition over extended propagation lengths and then adjust free electron density distribution to be intense, long and uniform through photo–electron interactions.

**Figure 23:**
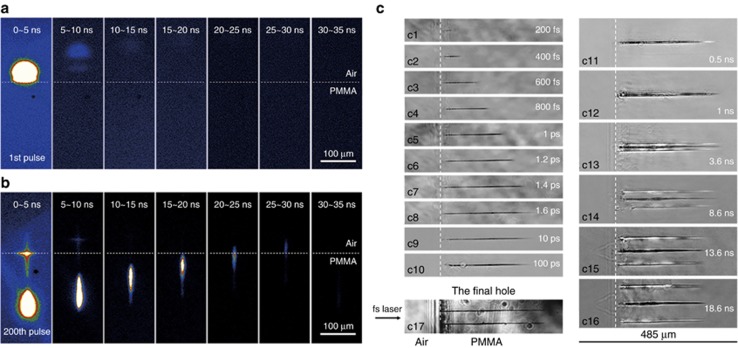
Time-resolved images of the plasma expansion during the Gauss beam drilling for the 1st pulse (**a**) and 200th pulse (**b**), respectively^133^, pump-probe shadowgraph study (**c**) of Bessel beam drilling^131^. **c**1–**c**16 Time-resolved images of femtosecond-picosecond-nanosceond dynamics of Bessel beam drilling. **c**17 The final hole morphology drilled by Bessel beam. Reproduced from Ref. 133 (with the permission of OSA) and Ref. 131 (with the permission of OSA).

Using the aforementioned multiscale time-resolved measurement system developed by us, we further revealed the forming mechanisms of the high aspect-ratio and high quality microholes using a femtosecond laser spatial pulse shaping beam (Bessel beam) by comparing it with the Gaussian beam percussion drilling. Figure 23a shows the time-resolved plasma photography of a Gaussian beam drilling with a single-pulse, the ablation plume was detected only above the PMMA surface. As shown in Figure 22c, the focused Gaussian beam was condensed, thus, the laser energy could only be localized within several microns below the surface. Such a high-energy concentration would make free electron density much higher than the critical density. Therefore, the Coulomb explosion and electrostatic ablation are weakened, and the material removal is mainly attributed to melting and evaporation, resulting in a large heat-affected zone. Meanwhile, the shielding-effect induced by the ultrahigh free electron density suppresses the laser propagation, leading to a short optical penetration depth; consequently, only a shallow crater can be fabricated with a single-pulse Gaussian beam. Furthermore, during the multipulses percussion drilling process (Figure 23b), the subsequent pulses energy was not sufficiently absorbed by the material because of the strong reflection of the generated dense ablated plasma. The generated plasma would disturb the propagation of the subsequent laser pulses so that leads to an unstable filament bending slightly deep inside the hole, then the laser beam energy was deposited along a bent direction. Moreover, for the deeper microholes, the plasma would cool down gradually, then adhered to the microholes sidewalls, which negatively affected the quality of the microholes.

By contrast, spatial shaping pulse (Bessel beam) drilling exhibits a completely different fabrication mechanism. Figure 23c displays the time-resolved images of Bessel beam drilling in PMMA. As the Bessel beam entered into the PMMA, the rising edge induced electron excitation, which formed a dark strip along the light path that indicated the plasma channel dynamics. The maximum length (optical penetration depth) of the Bessel plasma channel was reached at 1.6 ps and effectively coincided well with the final hole-depth, indicating the dominant role of initial electron excitation and the free electron distribution on the final structure formation. In the following tens of picoseconds, there existed electron-ion energy transfer occurred that induced an extremely high pressure and temperature in the focal area, resulting in an explosive and supersonic expansion of the material in the nanosecond domain, as shown in Figure 23c11. In contrast to the pressure waves in air and some other cases, the pressure wave induced by the Bessel beam in PMMA was a cylindrical shockwave and gradually expanded outward along the radial direction. This phenomenon suggests that the formation of microholes is an extrusion effect, leading to the formation of high quality microholes with taper-free, reduced recast/ejected materials and minimized in-cavity residues.

**Figure 24:**
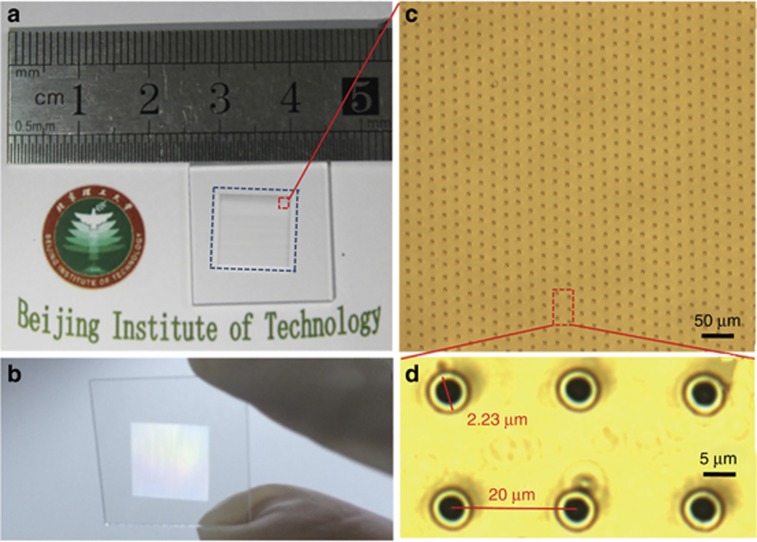
(**a** and **b**) The image of a microhole array throughout a 1 cm × 1 cm large area using the flying punch method for Bessel beam; (**c**) the local magnified OTM image of a microhole array; (**d**) the local magnified OTM image of microholes in the central array under 100 × microscope objective. Reproduced from Ref. 132 (with the permission of Springer).

After the understanding of the processing mechanisms of the Bessel beam, we proposed the flying punch method for machining large-area microhole arrays in PMMA by using a Bessel beam, as shown in Figure 24. A 1 cm × 1 cm microhole array (with 251 001 ultrahigh-aspect-ratio holes in total, at a processing speed of 100 holes per second) was fabricated within 42 min, indicating the high-efficiency and high repeatability of the fabrication process using the flying punch method^132^.

## Conclusions and outlooks

In this paper, we comprehensively reviewed our decade-long efforts on four parts of EDC in femtosecond laser micro/nano fabrications: the theoretical fundamentals, experiments, multiscale measurements and applications. Theoretically, based on the four models with different time scales (10^−3^–10^−15^ s) and space scales (10^−3^–10^−10^ m), we demonstrated that the localized transient electron dynamics (including electron density, temperature and excited state distribution), and subsequent phase change can be controlled by temporally/spatially shaping femtosecond laser pulses. Experimentally, seven experiments were reported as examples to validate the feasibility of EDC by temporally/spatially shaping femtosecond pulses in micro/nanofabrication. The experiments revealed that the precisions, efficiencies and qualities can be significantly improved and that various surface micro/nano-structures can be effectively modulated by the proposed EDC-based methods. Additionally, multiscale measurements further directly demonstrated the fundamentals of EDC from femtosecond scale to nanosecond scale and to millisecond scale. Finally, EDC was applied in high aspect-ratio (330:1) and high-quality microholes drilling at the speed of 100 holes per second (251 001 holes fabricated in 1 cm × 1 cm area within 42 min), in which multiscale measurements were used to analyze and optimize the electron dynamics. The high aspect-ratio microholes drilling was applied to key structure fabrication in one of the 16 Chinese National S&T Major Projects.

We have devoted the past ten years to studying the mechanisms, methodologies and applications of femtosecond laser micro/nano fabrications. However, many challenges still remain, especially on the following topics:

1. Comprehensive models: Femtosecond laser-material interactions are a comprehensive nonlinear, nonequilibrium process ranging from a nanometer scale to a millimeter scale and from a femtosecond scale to a millisecond scale. However, our present models, including the plasma and improved two-temperature models, are not applicable to some materials. Furthermore, a comprehensive, integrated multiscale physical-chemical modeling, from a nanometer scale to a millimeter scale and from a femtosecond scale to a microsecond scale, shall be developed and improved to describe femtosecond laser-material interactions. Attosecond laser-material interactions shall also be studied.
2. Method improvements: In our previous work, by designing and shaping femtosecond laser pulses in temporal/spatial domain(s) to adjust localized transient electron dynamics, the throughput, quality and aspect-ratio limit was greatly improved/extended. However, we shall: (i) further optimize the experiment parameters to enhance/improve/extend the fabrication efficiency, quality and limit; and (ii) employ these novel fabrication methodologies in other fabrication areas, such as three-dimensional laser bio-printing fabrication, green energy fabrication and biomimetic materials fabrication.
3. Multiscale measurement system improvements: By using the current multiscale measurement system, the panoramic dynamics of laser ablation from a femtosecond scale to a second scale have been revealed. However, the intrinsic characteristics of a probe beam (wavelength and pulse duration) and the signal collecting system limited the spatial/temporal resolution and the sensitivity of the measurement, which shall be improved to monitor the electron dynamics in more detail. In addition, more time-resolved measurement techniques should be integrated into the multiscale system, so that it can provide more information of electrons from different aspects. Furthermore, the measurement results should correspond with the theoretical models, such as providing the characteristic values of electrons for theoretical calculations.
4. Broader applications: High-aspect-ratio and high-quality microholes by using spatial shaping pulses have been applied in fabricating some key structure fabrications. However, the current method is limited to a few transparent materials. We will extend the range of materials and then explore novel applications of microholes, such as for microfluidic devices and three-dimensional integrated chip packaging. In addition, we will substantially expand the applications of the novel method, such as for the adjustment of chemical reaction pathways by ultrafast laser EDC, transient or permanent adjustment of material properties through ultrafast laser EDC.

## Figures and Tables

**Table 1:** The estimations of optical and thermal properties in classical and improved two-temperature models^116^

Property	Classical TTM	Improved TTM
Electron heat capacity	*C*_e_=*γT*_e_	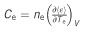
Electron relaxation time	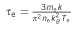	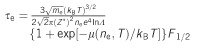
Electron heat conductivity	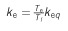	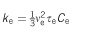
Optical property	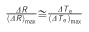