# Supplementary material for: Electrons dynamics control by shaping femtosecond laser pulses in micro/nanofabrication: modeling, method, measurement and application
Source: Light Sci Appl. 2018 Feb 9;7:17134–. doi: 10.1038/lsa.2017.134 (PMC6060063; doi:10.1038/lsa.2017.134)
Supplement: Supplementary Material [file lsa2017134x1.docx]

Supplementary Information for

Electrons Dynamics Control by Shaping Femtosecond Laser Pulses in Micro/Nano Fabrication: Modeling, Method, Application, and Measurement

Contents

Movie 1. Concept of Electrons Dynamics Control.

Movie 2. Focused femtosecond laser propagation in PMMA in femtosecond-to-picosecond time scale

Movie 3. Plasma and shockwave expansion in picosecond-to-nanosecond time scale during the laser interaction with PMMA

Movie 4. High aspect-ratio micro-hole formation in PMMA

Movie 5. Plasma ejection on the surface and within the hole

Movie 6. Plasma ejection in nanosecond time scale during the laser interaction with PMMA
