# Supplementary figures and images for: Electrons dynamics control by shaping femtosecond laser pulses in micro/nanofabrication: modeling, method, measurement and application
Source: Light Sci Appl. 2018 Feb 9;7:17134–. doi: 10.1038/lsa.2017.134 (PMC6060063; doi:10.1038/lsa.2017.134)

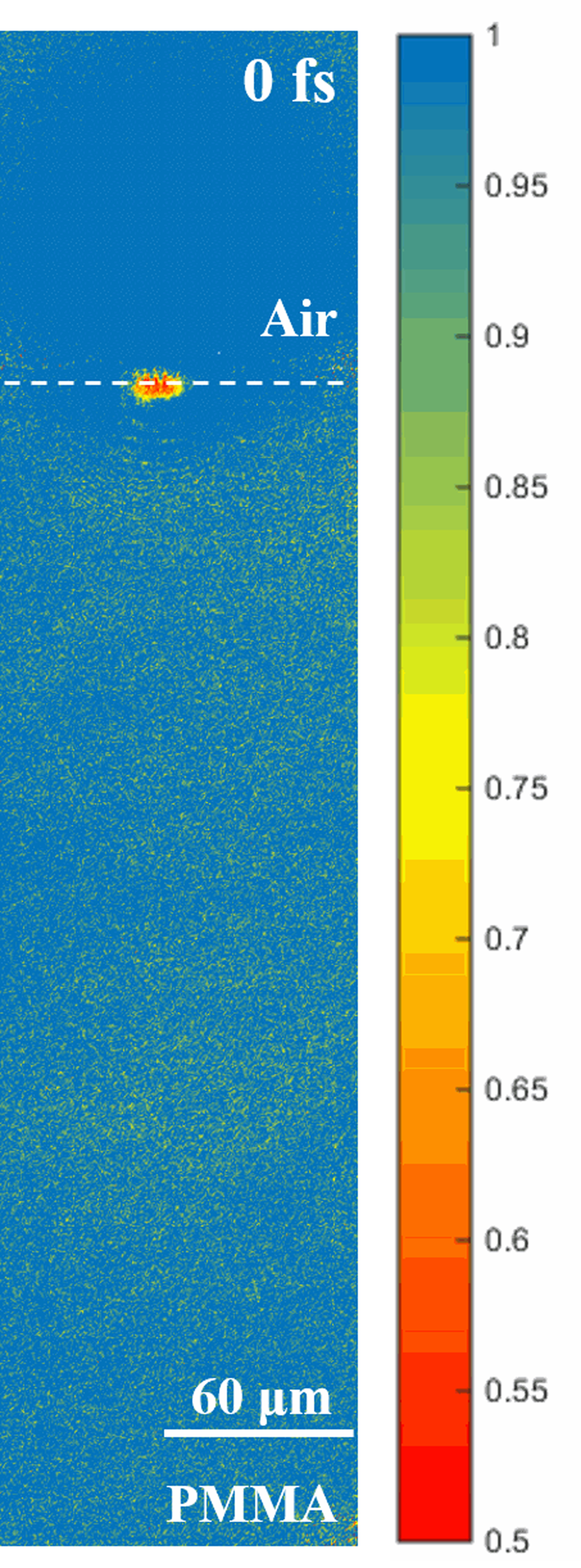

Supplement: Supplementary Movie 2 [file lsa2017134x3.tif]

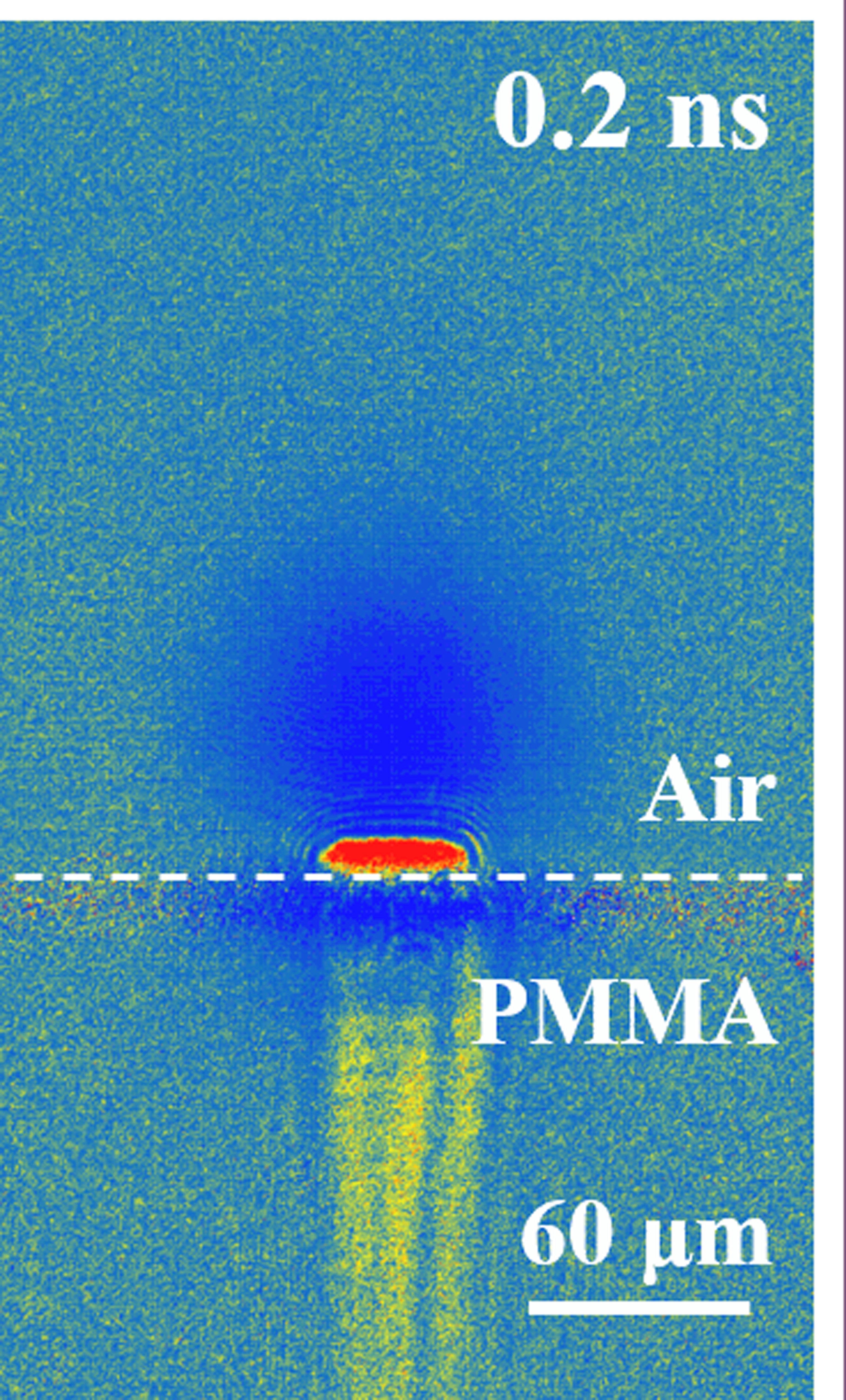

Supplement: Supplementary Movie 3 [file lsa2017134x4.tif]

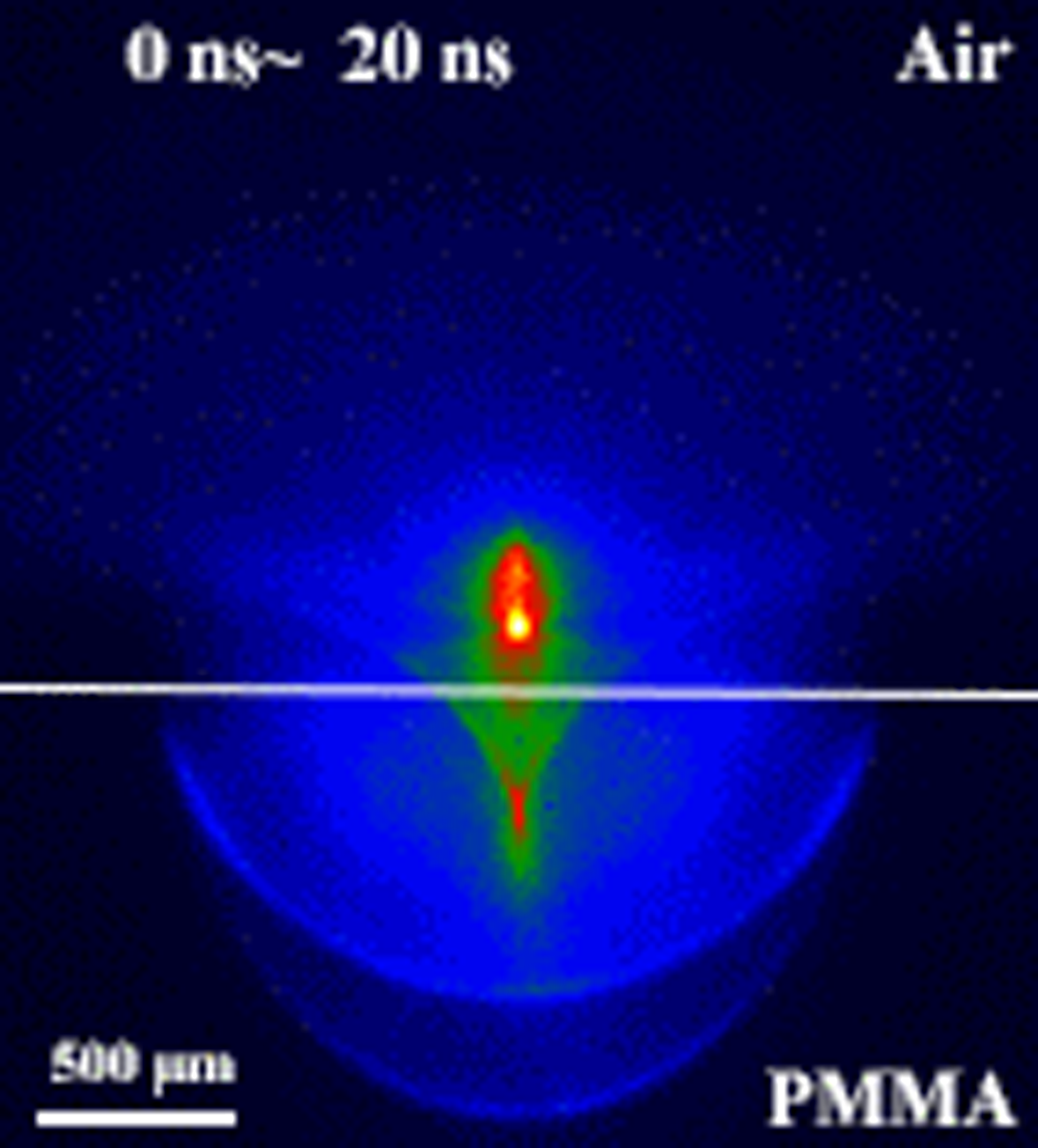

Supplement: Supplementary Movie 4 [file lsa2017134x5.tif]

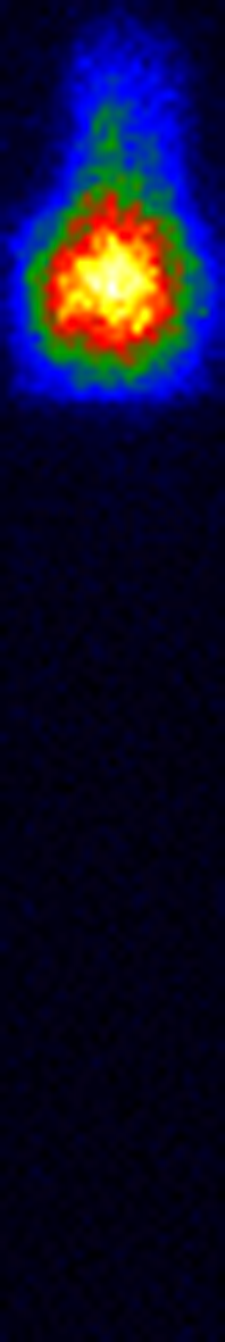

Supplement: Supplementary Movie 5 [file lsa2017134x6.tif]

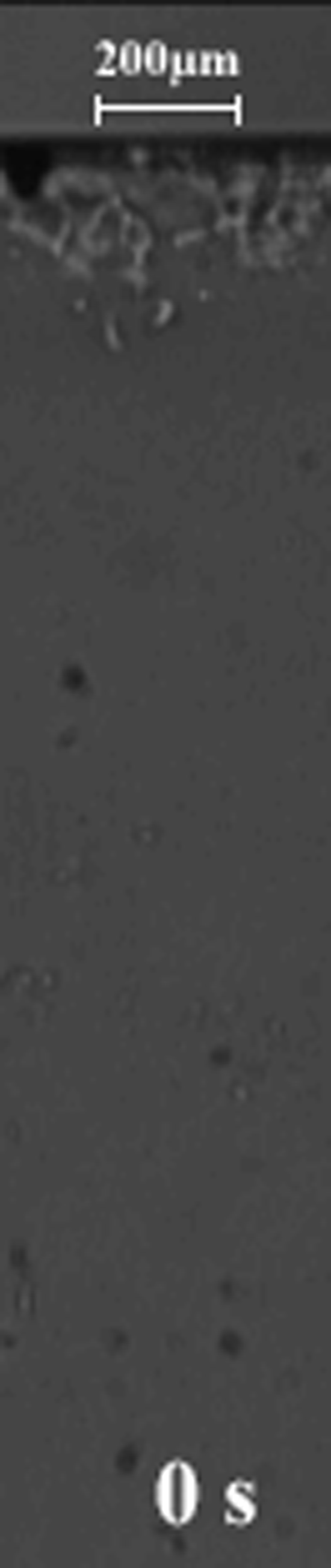

Supplement: Supplementary Movie 6 [file lsa2017134x7.tif]
